# Culture-Based and Culture-Independent Assessments of Endophytic Fungal Diversity in Aquatic Plants in Southwest China

**DOI:** 10.3389/ffunb.2021.692549

**Published:** 2021-07-27

**Authors:** Hua Zheng, Min Qiao, Jianping Xu, Zefen Yu

**Affiliations:** ^1^Laboratory for Conservation and Utilization of Bio-Resources, Key Laboratory for Microbial Resources of the Ministry of Education, Yunnan University, Kunming, China; ^2^School of Life Sciences, Yunnan University, Kunming, China; ^3^Department of Biology, McMaster University, Hamilton, ON, Canada

**Keywords:** high-throughput sequencing, aquatic plant, endophytic fungi, fresh water, fungal composition and diversity

## Abstract

Aquatic ecosystems contain tremendous plant and microbial diversity. However, little is known about endophyte diversity in aquatic plants. In this study, we investigated the diversity of endophytic fungi in aquatic plants in southwest China using both culture-based and culture-independent high-throughput sequencing methods. A total of 1,689 fungal isolates belonging to three phyla and 154 genera were obtained from 15,373 plant tissue segments of 30 aquatic plant species. The most abundant endophytic fungi were those in ascomycete genera *Aspergillus, Ceratophoma, Fusarium, Penicillium, Phoma* and *Plectosporium*. No difference in fungal isolation rates was observed among tissues from roots, stems, and leaves. Twenty tissue samples from three most common plant species were further subjected to culture-independent meta-barcode sequencing. The sequence-based analyses revealed a total of 1,074 OTUs belonging to six fungal phyla and 194 genera. Among the three plants, *Batrachium bungei* harbored the highest number of OTUs. Besides, a total of 66 genera were detected by two methods. Both the culture-dependent and independent methods revealed that aquatic plants in southwest China have abundant endophytic fungal diversity. This study significantly expands our knowledge of the fungal community of aquatic plants.

## Introduction

Fungi are heterotrophic eukaryotes that are ubiquitous on earth, inhabiting diverse terrestrial, marine, and freshwater environments (Hawksworth, 2001; Blackwell, 2011). They play major roles as decomposers, form essential associations with other organisms, and are among the major drivers in nutrient cycling and soil formation (Blackwell, 2011). Currently, about 140,000 fungal species have been reported worldwide (https://www.catalogueoflife.org/, May 2021), but recent study estimated 2.2 to 3.8 million fungal species may exist on Earth (Hawksworth and Lücking, 2017). The vast majority of fungi are still unknown, and many of these are likely to occur in previously underexplored environments.

Some fungi live in the tissues and organs of healthy plants at a certain stage or all stages of plant's life cycle and cause no symptomatic infections. These fungi are known as endophytes and they are a significant part of the plant microbiome. Furthermore, these fungal communities associated with plants are not randomly assembled (Bulgarelli et al., 2012), and previous studies have demonstrated the role played by the plant in the recruitment of a particular microbial consortium to adapt itself to the environmental conditions (Lê Van et al., 2017; Illescas et al., 2020). As a group, endophytes interacted with hosts to perform a range of associations and functions, from defensive mutualism and enhancement of stress tolerance to latent pathogenicity (Carroll, 1988; Arnold, 2007; Rodriguez et al., 2009; Bacon and White, 2016). In addition, many studies have reported that endophytes frequently produce diverse secondary metabolites, and many of which are important in agriculture, industry, and medicine (Porras-Alfaro et al., 2011; Giauque and Hawkes, 2013; Sandberg, 2013; Gupta et al., 2020).

Despite growing interests in the ecology, evolution, and applications of endophytes, relatively little is known about the geographic and ecological distributions of endophytes in most plant communities. Moreover, the vast majority of endophyte studies have focused on plants in terrestrial systems (Rodrigues, 1994; Faeth and Hammon, 1997; Arnold et al., 2001; Arnold and Lutzoni, 2007; U'Ren et al., 2010; Massimo et al., 2015; Gao et al., 2019). In contrast, endophytes in plants of the marine and freshwater ecosystems have received very little attention thus far.

More than 70% of the earth's surface is covered by water, as oceans, seas, ponds, rivers, and lakes (Polunin, 2008). Plant communities inhabiting freshwater systems, which are usually described as aquatic plants, include phylogenetically diverse vascular plants. Many aquatic plants die back in winter in strongly seasonal sites, with new growth initiated in spring from overwintering shoots and roots (Shearer, 2001). Aquatic plants play a significant role in maintaining water quality, removing excessive nutrient load, absorbing nutrient mineral ions and reducing sediment resuspension (Srivastava et al., 2008; Ong et al., 2010; Baldy et al., 2015). According to growth forms, aquatic plants were usually classified into five types: emergent plants (e.g., *Sagittaria trifolia* L.), floating-leaved plants (e.g., *Potamogeton wrightii* Morong, *Nymphoides peltatum* (Gmel.) O.Kuntze), free-floating plants (e.g., *Eichhornia crassipes* (Mart.) Solms, *Pistia stratiotes* L.), submerged plants (e.g., *Myriophyllum spicatum* L., *Ceratophyllum demersum* L.), and wet plants (e.g., *Polygonum amphibium* L.). Many researchers have investigated the diversity of aquatic plants and revealed their ecological function (Bornette et al., 1998; Ansari et al., 2010; Kumar et al., 2020), but the impact of their endophytes on host plants and on their broad ecological functions are still largely ignored. Indeed, compared to the large number of studies on endophytes of terrestrial plants, there are very few studies on the diversity, distributions, host affiliations, or tissue preferences of endophytes in aquatic plants (Sati and Belwal, 2005; Bornette and Puijalon, 2011; Sandberg et al., 2014). In addition, previous studies on endophytic fungi in aquatic plants have focused on few species of aquatic plants and/or sampling sites (Li et al., 2010; Kohout et al., 2012; Sandberg et al., 2014; You et al., 2015).

Traditionally, cultivation dependent techniques have been employed in endophyte diversity studies (Petrini et al., 1982; Sun et al., 2011; Chowdhary and Kaushik, 2015; Teasdale et al., 2018). Such studies are important to specifically characterize the individual isolated endophytic fungi, including testing their potential for being as inoculants to improve plant growth and health, or screening for novel biologically active secondary metabolites (Ding et al., 2009; Tejesvi et al., 2011; Khan et al., 2016; Depetris et al., 2020; Zhao et al., 2020). Nevertheless, the isolation of endophytic fungi has been limited by traditional methodology due to our inability to grow many fungi in the laboratory. Fortunately, the development of molecular biology brings a new perspective to endophyte diversity studies. Specifically, high-throughput DNA sequencing technologies can provide abundant information for analyzing the community composition of endophytic fungi, including many difficult to culture or unculturable fungi. Over the last decade, there have been rapid progresses in fungal biodiversity research using the cultivation-independent high-throughput sequencing technologies (Kemler et al., 2013; Sun et al., 2016; Schmidt et al., 2018; Bataineh et al., 2020). Among the high-throughput DNA sequencing platforms, the Illumina MiSeq and HiSeq platforms are the most commonly used. They are capable of producing tens of millions of reads and multiplex hundreds of samples in a single run (Caporaso et al., 2011; Smith and Peay, 2014). These platforms have been used to analyze fungal diversity in a range of samples, from stored dates (Al-Bulushi et al., 2017) to sour soup (Lin et al., 2020).

Southwest China is one of the world's 34 biodiversity hotspots (Myers et al., 2000). During ongoing exploration for microfungi inhabiting freshwater systems in this area, several new species have been described in recent years (Guo et al., 2019; Qiao et al., 2020; Zheng et al., 2020a,b, 2021a,b). This region covers three provinces, Guizhou, Yunnan, and Sichuan. Sichuan province has the average altitude 500 meters, is made up of a basin and surrounded by mountains. Yunnan and Guizhou provinces have average elevations of 2,000 and 1,000 m, respectively, and with high mountains and deep valleys distributed throughout both provinces. All three provinces have a subtropical monsoon climate, with lush and abundant endemic and shared vegetations in many regions within each province (Zhang et al., 2011). Due to their geographic features, each province also has many lakes, rivers and wetlands.

Due to the important roles of aquatic plants in the aquatic ecosystems and the potential impacts of endophytic fungi on aquatic plants, it is essential to understand the fungal diversity in aquatic plants. In order to understand the endophytic fungi diversity of aquatic plants in Southwest China, 30 aquatic plant species were collected from lakes, rivers, ponds, reservoirs and Wetlands in Yunnan, Guizhou and Sichuan provinces in this study. We assessed the diversity, host affiliations, and geographic distributions of endophytic fungi associated with aquatic plants by using both culture-dependent and culture-independent methods.

## Materials and Methods

### Plant Sample Collection and Surface Sterilization

**Figure 1:**
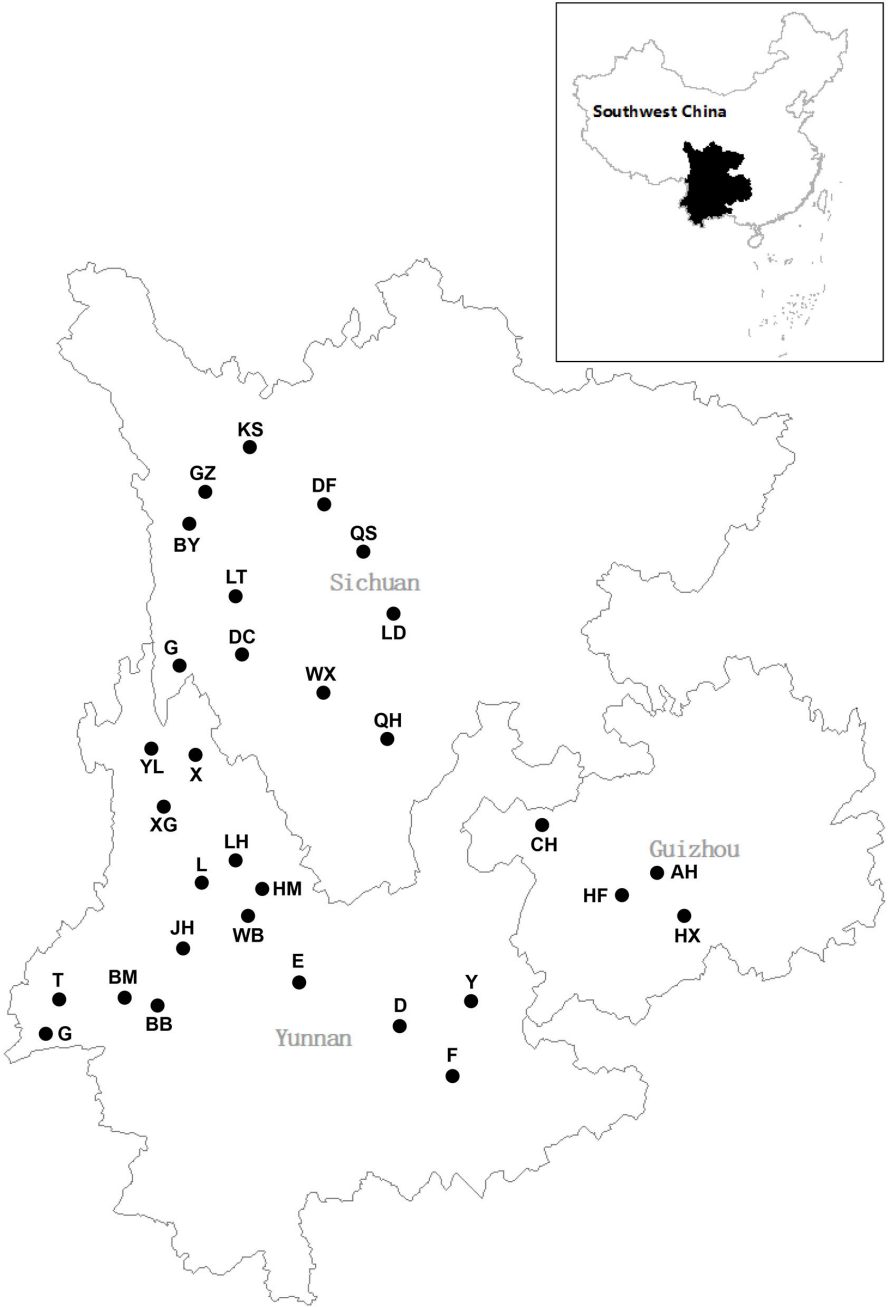
Distribution of sampling sites.

**Figure 2:**
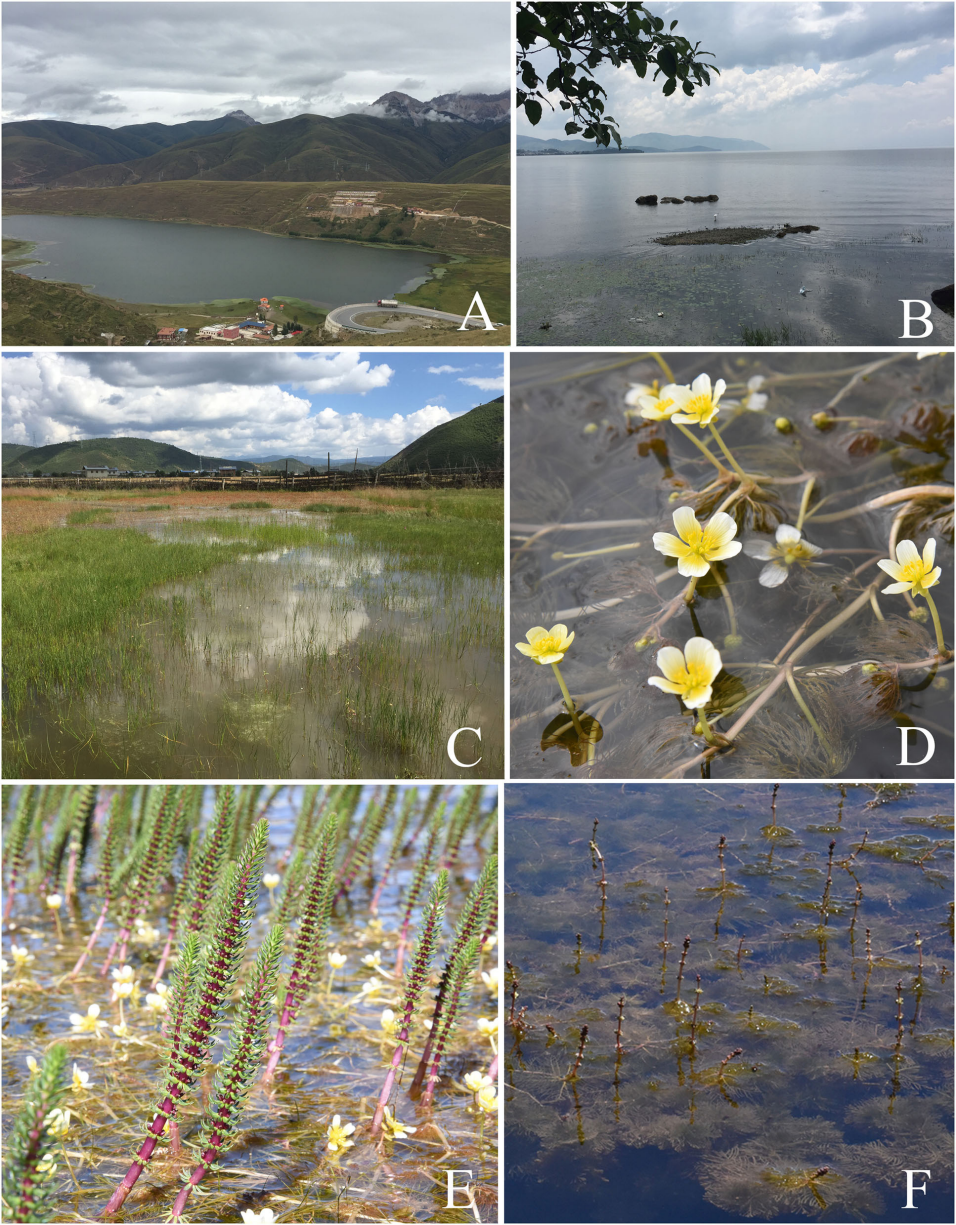
Representative sampling sites and aquatic plants. **(A)** The sampling site Kashahu in Sichuan Province. **(B)** The sampling site Erhai in Yunnan Province. **(C)** The sampling site Xianggelila in Yunnan Province. **(D)** The aquatic plant *Batrachium bungei*. **(E)** The aquatic plant *Hippuris vulgaris*. **(F)** The aquatic plant *Myriophyllum spicatum*.

Plant samples were collected from 16 sampling sites in Yunnan Province between July and September 2015, four sampling sites in Guizhou province (July), and 11 sampling sites in Sichuan province (August–September) (Figure 1, Supplementary Table 1). Thirty plant species belonging to 16 families were collected from freshwater environments (Figure 2; Table 1). Healthy, mature plants having undamaged leaves were fully uprooted and brought to the laboratory in sterile polythene bags. During transport, plant samples were stored at 4°C.

**Table 1:** Family and species information of the 30 aquatic plant species collected from Southwest China.

**Family**	**Species**	**Type**
Alismataceae	*Sagittaria trifolia* L.	Emergent plant
	*Alternanthera philoxeroides* (Mart.) Griseb.	Wet plant
Araceae	*Pistia stratiotes* L.	Free-floating plant
Ceratophyllaceae	*Ceratophyllum demersum* L.	Submerged plant
Gentianaceae	*Nymphoides peltatum* (Gmel.) O.Kuntze	Floating-leaved plant
Guttiferae	*Elodea canadensis* Michx.	Submerged plant
Haloragaceae	*Myriophyllum spicatum* L.	Submerged plant
	*Myriophyllum sibiricum* Komarov	Submerged plant
	*Myriophyllum aquaticum* (Vell.) Verdc.	Emergent or submerged plant
	*Hippuris vulgaris* L.	Wet plant
Hydrocharitaceae	*Hydrilla verticillata* (Linn. f.) Royle	Submerged plant
	*Vallisneria natans* (Lour.) Hara	Submerged plant
	*Hydrocharis dubia* (Bl.) Backer	Floating-leaved plant
	*Egeria densa* Planch.	Submerged plant
	*Ottelia acuminata* (Gagnep.) Dandy	Submerged plant
Isoetaceae	*Isoetes sinensis* Palmer	Wet plant
Leguminosae	*Medicago sativa* L.	Wet plant
Polygonaceae	*Polygonum amphibium* L.	Wet or emergent plant
Pontederiaceae	*Eichhornia crassipes* (Mart.) Solms	Floating-leaved plant
Potamogetonaceae	*Potamogeton distinctus* A.Benn.	Floating-leaved or submerged plant
	*Potamogeton wrightii* Morong	Floating-leaved or submerged plant
	*Potamogeton pectinatus* L.	Submerged plant
	*Potamogeton perfoliatus* L.	Submerged plant
	*Potamogeton lucens* L.	Submerged plant
	*Potamogeton oxyphyllus* Miq.	Submerged plant
	*Potamogeton acutifolius* Roem. et Schult.	Submerged plant
	*Potamogeton intortifolius* J. B. He et al.	Submerged plant
Ranunculaceae	*Batrachium bungei* (Steud.) L. Liou	Submerged plant
Trapaceae	*Trapa bispinosa* Roxb.	Floating-leaved plant
	*Trapa natans* L.	Floating-leaved plant

In the laboratory, each plant sample was rinsed for 30 s in running tap water, dried gently with paper towels. Then, plant samples were processed using the following sequential steps: initial immersion for 2 min in 0.5% sodium hypochlorite, followed by 1 min in sterile distilled water, 2 min in 75% ethanol, and finally 1 min in sterile distilled water. After the final wash, the plant materials were dried using sterile paper towels. These surface-sterilized plants were partitioned into roots, stems and leaves. In addition, the tissue samples were divided into two parts and placed individually into 1.5 mL microcentrifuge tubes, one part to be used for fungal culturing and the other for meta-barcode Illumina sequencing.

### Isolation, Culture and Identification of Cultivable Endophytic Fungi

In our first study of these aquatic plants' cultivable endophytic fungi, these surface-sterilized plants were cut into ca. 5 ×5 mm segments, and then these tissue segments, including roots, stems and leaves, were placed on rose bengal agar (RBA; peptone 5.0 g, dextrose 10.0 g, potassium dihydrogen phosphate 1.0 g, magnesium sulfate 0.5 g, Agar 15.0 g, rose bengal 0.033 g, chloramphenicol 0.1 g, 1 L distilled water) medium amended with penicillin G (0.5 g/L) and streptomycin (0.5 g/L) (Gams et al., 1998). The plate tissues were incubated at 25°C and checked every other day for 21 days. Fungal mycelia growing out from the plant tissues were transferred to other plates containing potato dextrose agar (PDA; potato 200 g, glucose 20 g, agar 18 g, 1 L distilled water). The efficacy of the sterilization procedure was confirmed by the method of Schulz et al. (1998).

When all 1,689 pure fungal isolates were obtained, the total DNA of each isolate was extracted from fresh fungal mycelia grown on PDA plates, following the protocol of Turner et al. (1997). The nucleotide sequence of the internal transcribed spacer (ITS) regions of ribosomal DNA of the obtained fungi was amplified using primers ITS1 and ITS4 (White et al., 1990). The PCR conditions were pre-denaturation (94°C, 4 min), denaturation (94°C, 1 min), annealing (55°C, 1 min), and extension (72°C, 2 min) for a total of 35 cycles, followed by a final extension (72°C, 2 min). The obtained ITS sequences (Supplementary Table 2) were compared with those in GenBank using BLAST searches to determine the taxonomy of these fungi, mostly to the genus level.

### Analysis of Cultivable Endophytic Fungi

The endophytic fungi isolation frequency was calculated as the total number of fungal endophytes divided by the total number of incubated plant tissue segments multiplied by 100 (Petrini and Fisher, 1988), reflecting the number of fungi per fragment of different plants. The endophytic fungal diversity was evaluated using the Shannon index (H′), which has two main components, evenness and richness of the fungal species. H′ was derived according to following equation: – Σ (Pi ln [Pi]); where Pi = ni/N, ni = number of individuals of the species i, and N = total number of individuals of all species (Pielou, 1975). The Pielou evenness was obtained using the following equation: J′ = H′/Log (S), where H′ is the value calculated by the Shannon index and S is species richness. Evenness expresses how evenly the individuals in the community are distributed among species. Species richness (S), the total number of unique species, is the simplest measure of biodiversity. To evaluate the degree of community similarity of isolated fungal endophytes between different altitudes, Sorenson's coefficient similarity index (CS) and Jaccard's similarity coefficient index (CJ) were employed and calculated according to the following formula: *CS* = 2j/(a +b), CJ = j/(a +b-j); where j is the number of endophytic fungal species co-existing in different elevations, a is the total number of endophytic fungal species in one altitude condition, b is the total number of endophytic fungal species in another altitude condition (Su et al., 2010).

### Molecular Detection of Endophytes by Illumina MiSeq Sequencing

We selected the most abundant plant species *Batrachium bungei* (Steud.) L. Liou to analyze the potential difference of endophytic fungi in this plant among different sampling sites, including at different elevations and types of sampling sites, by Illumina MiSeq sequencing. In addition, we compared endophytic fungi among three aquatic plants: *M. spicatum* L., *Hippuris vulgaris* L., and *B. bungei* collected from the same lake. For each of these comparisons, surface-sterilized tissue samples were ground in liquid nitrogen using a mortar and pestle. All plant samples were divided into root, stem, and leaf for sequencing, and there is no biological duplication. A total of 20 samples were used for culture-independent analysis (Table 2). The total DNA of these plant samples were extracted using the DNeasy Plant Mini Kit (Qiagen) according to the manufacturer's recommendations. The sample DNA concentration and quality were examined using 1% agarose gel electrophoresis and a NanoDrop One spectrophotometer (Thermofisher Scientific, USA). Using 20–30 ng DNA of each sample as template, the ITS1 variable region was amplified using primers ITS1 (5′-CTTGGTCATTTAGAGGAAGTAA-3′) and ITS2 (5′-GCTGCGTTCTTCATCGATGC-3′) (Sun et al., 2018). Each PCR was performed in triplicates in a 25 μL mixture containing 2.5 μL of Trans Start buffer, 2 μL of dNTPs, 1 μL of each primer, and 20 ng of template DNA, which proceeded under the following parameters: initial denaturation at 94°C for 5 min, followed by 25 cycles of denaturation at 94°C for 30 s, annealing at 57°C for 30 s, elongation at 65°C for 30 s, and a final extension at 72°C for 5 min. The PCR products were detected using 1.5% agarose gel electrophoresis. The sequencing libraries were then built, with each sample ligated to a different linker barcode sequence. The validated DNA library concentrations were assessed using a Qubit 3.0 fluorometer. After adjusting the library volume based on the target data volume and mixing the multiple libraries on an Illumina MiSeq sequencing instrument (Illumina, San Diego, CA, USA), sequencing was performed using PE250 paired-end sequencing. Image analysis and base calling were conducted by the MiSeq control software (MCS) embedded in the MiSeq instrument. The data generated from the Illumina sequencing samples was deposited in the National Center for Biotechnology Information Sequence Read Archives (SRA) as BioProject ID PRJNA736183.

**Table 2:** The detail information of samples for high-throughput sequencing.

**Sample**	**Tissue**	**Plant species**	**Sampling Site**	**Type**	**Elevation (m)**
D_s_g	Root	*Batrachium bungei*	Daocheng (DC)	Wetland	4,053
D_s_J	Stem				
D_s_Y	Leaf				
H_s_g	Root	*Hippuris vulgaris*	LitangII (LTII)	River	3,886
H_s_J	Stem				
H_s_Y	Leaf				
K_s_g	Root	*Batrachium bungei*	Kashahu (KS)	Lake	3,505
K_s_J	Stem				
K_s_Y	Leaf				
L_s_g	Root	*Batrachium bungei*	KashahuII (KSII)	Pond	3,992
L_s_J	Stem				
L_s_Y	Leaf				
S_J	Stem	*Myriophyllum spicatum*	LitangII (LTII)	River	3,992
S_Y	Leaf				
W_s_g	Root	*Batrachium bungei*	LitangII (LTII)	River	3,992
W_s_J	Stem				
W_s_Y	Leaf				
X_s_g	Root	*Batrachium bungei*	Xiajisha (XG)	Wetland	3,275
X_s_J	Stem				
X_s_Y	Leaf				

### Data Analysis for Illumina MiSeq Sequencing

High-throughput sequencing was performed using Illumina MiSeq PE300 platform by Majorbio Bio-Pharm Technology Co. Ltd. (Shanghai, China), then, the data were analyzed on the free online platform of Majorbio Cloud Platform (www.majorbio.com). The raw sequencing reads were demultiplexed, quality-filtered by fastp version 0.20.0 (Chen et al., 2018) and merged by FLASH version 1.2.7 (Magoč and Salzberg, 2011) with the following criteria: (i) the 300 bp reads were truncated at any site receiving an average quality score of <20 over a 50 bp sliding window, and the truncated reads shorter than 50 bp were discarded, reads containing ambiguous characters were also discarded; (ii) only overlapping sequences longer than 10 bp were assembled according to their overlapped sequence. The maximum mismatch ratio of overlap region is 0.2. Reads that could not be assembled were discarded; (iii) Samples were distinguished according to the barcode and primers, and the sequence direction was adjusted, exact barcode matching, 2 nucleotide mismatches in primer matching. Operational taxonomic units (OTUs) with 97% similarity cutoff (Stackebrandt and Goebel, 1994; Edgar, 2013) were clustered using UPARSE version 7.1 (Edgar, 2013), and chimeric sequences were identified and removed. OTUs represented by fewer than 5 sequences were removed, as these OTUs tend to be artifactual (Lindahl et al., 2013). The taxonomy of each OTU representative sequence was analyzed by RDP Classifier version 2.2 (Wang B. et al., 2007; Wang Q. et al., 2007) against the UNITE ITS database (https://unite.ut.ee/) using confidence threshold of 0.7. The community composition of each sample was counted at the kingdom, phylum, class, order, family, genus and species levels. After obtaining the sequencing result and calculation of OTUs matrix, Qiime (V. 1.9.1) was used for full-sample similarity comparison to analyze the alpha-diversity and calculate the observed-species, Chao1, Shannon, Simpson, ACE, and Good's-coverage indices (Schloss et al., 2009; Caporaso et al., 2010). R software (Version 2.15.3) was used to draw a dilution curve. Each OTU was assigned to a functional guild using the FUNGuild database (http://www.funguild.org/).

## Results

### Culture-Dependent Fungal Diversity and Abundance

A total of 1,689 fungal isolates were isolated from 15,373 tissue segments of thirty aquatic plant species. Based on ITS sequence data, these isolates were classified into 154 genera, including 123 genera Ascomycota, 29 genera Basidiomycota, 2 genera Zygomycota, respectively. The generic distribution of the 1,697 isolates is presented in Supplementary Table 2. The most abundant endophytic fungi of all aquatic plants were those in genera *Aspergillus, Ceratophoma, Fusarium, Penicillium, Phoma* and *Plectosporium* in Ascomycota.

We calculated the isolation frequency, Shannon index, and Pielou evenness of different aquatic plants, to compare the richness and diversity of endophytic fungi among samples. The results are shown in Supplementary Table 3. The average of fungal isolation frequency of all aquatic plant samples was 11%, and the three tissue types (root, stem, and leaf) did not differ significantly in the cultivable fungi isolation frequency. The plant species that showed higher than average fungal isolation frequencies are those plant species *Pistia stratiotes* (11.3%)*, Vallisneria natans* (11.4%), *M. aquaticum* (11.4%), *M. sibiricum* (12.0%), *Potamogeton acutifolius* (13.0%), *P. intortifolius* (14.0%), *H. vulgaris* (13.2%), *Isoetes sinensis*2 (18.2%), *P. distinctus* (16.7%), *Eichhornia crassipes* (17.7%)*, Alternanthera philoxeroides* (19.1%) and *Hydrocharis dubia* (22.3%).

As the dominant plant species in different sampling sites are different, we counted the number of endophytic fungi isolated from all aquatic plants in each sampling sites, and obtained the isolation frequency and diversity index of different sampling sites. The detailed results are shown in Supplementary Table 4. Among the three provinces, the isolation frequencies differed slightly, with those from Yunnan province having an overall frequency of 13.6%, followed by Sichuan (9.4%) and Guizhou (9.2%). Among all sampling sites, the highest Shannon index was 4.43 in site D in Yunnan province, and the lowest is 2.16 in site XG also in Yunnan province. Moreover, the Pielou evenness was relatively high in all sampling sites (> 0.76), and most sampling sites exceeded 0.90. Altitudinally, there were differences among sites at different altitudes in both the species and the diversity of endophytic fungi in aquatic plants (Table 3). In general, the isolation frequency, species richness and diversity index of endophytic fungi tended to decrease as altitude increased. When analyzed for similarity index of endophytic fungi composition at different altitudes, the similarity of fungal composition is inversely proportional to the altitude difference (Table 4).

**Table 3:** General information of fungal endophytes isolated from sites at different altitudes.

**Elevation (m)**	**No. of isolates**	**No. of Genera**	**Isolation Frequency**	**Dominant Genera**	**H′**
1000–2000	466	88	13.15%	*Alternaria, Ceratophoma, PhomaFusarium, Plectosporium*	5.00
2000–3000	156	55	9.72%	*Phomopsis, PlectosporiumCytosporina, Phoma*	5.33
3000–4000	331	78	10.8%	*Ceratophoma, Phoma, Fusarium*	5.09
4000+	235	52	10.4%	*Alternaria, Phoma, Fusarium*	4.55

**Table 4:**
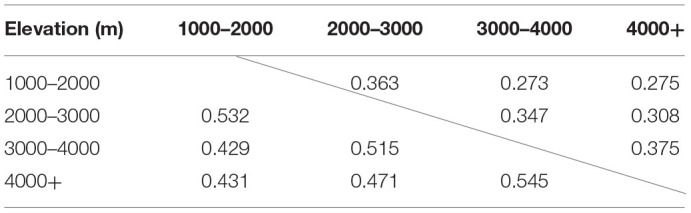
Similarity index of fungal endophytes isolated from different elevations at the genera level.

*Above the diagonal line in the table showed the Jaccard's similarity coefficient index in different elevation, and below the diagonal line was Sorenson's coefficient similarity index*.

### Sequence-Based Fungal Diversity

After quality checking for removing low quality, chimeric and rare sequences, a total of 1,049,080 high-quality sequences with an average length of 314 bp were obtained from 20 samples. These sequences were aggregated into 1,074 OTUs at a 97% similarity level. The rarefaction curve of the fungal community is shown in Supplementary Figure 1. When the number of sequences reached approximately 50,000, the Sobs index on OTU level tended to plateau, therefore, the sequencing depth was sufficient to cover all taxa in the 20 samples.

To analyze the fungal complexity in the plant samples, commonly used measures of diversity indices were calculated to infer fungal diversity and richness (Table 5), including ACE index, Chao index, Good's coverage, Shannon index and Simpson index. The Chao and ACE indexes indicate the taxa richness of the microbial community, while the Simpson and Shannon indexes represent the diversity of the microbial community (Soto Del Rio et al., 2017). According to these indexes, the diversity in host plant species *B. bungei* was higher than those in *H. vulgaris* and *M. spicatum*. Among different sampling sites, *B. bungei* collected at site KS had the richest fungal community, followed by those collected at site DC, site LTII, site XG and site KSII. However, the ACE and Chao1 indexes indicated that the root diversity of *B. bungei* sampled from site LTII was the highest among all samples, followed by site KS.

**Table 5:** Alpha- diversity indexes of fungal communities based on high-throughput DNA sequencing of different samples.

**Sample/Estimators**	**ACE**	**Chao**	**Coverage**	**Shannon**	**Simpson**
D_s_Y	385.282	380.466	0.999	2.114	0.311
W_s_g	534.813	535.216	0.999	3.084	0.163
H_s_Y	63.044	55.125	0.999	0.966	0.547
K_s_g	453.704	454.512	0.999	2.968	0.141
L_s_Y	41.472	40.000	0.999	0.054	0.987
W_s_J	198.187	196.687	0.999	1.617	0.524
D_s_g	231.267	232.035	0.999	1.230	0.448
K_s_Y	419.877	411.15873	0.998	0.659	0.811
W_s_Y	77.806	79.333	0.999	0.470	0.852
X_s_g	33.703	32.000	0.997	2.094	0.195
K_s_J	418.431	417.333	0.999	1.802	0.421
H_s_g	41.885	41.000	0.999	2.064	0.166
L_s_J	10.672	6.500	0.999	0.005	0.998
H_s_J	54.369	49.333	0.996	1.951	0.254
X_s_J	67.848	70.000	0.999	0.217	0.939
X_s_Y	115.640	117.300	0.999	0.334	0.908
S_Y	114.464	114.500	0.999	0.395	0.891
L_s_g	31.288	30.3750	0.999	0.152	0.942
S_J	115.490	114.750	0.999	1.419	0.580
D_s_J	319.517	322.800	0.999	1.066	0.671

*Richness and diversity estimation of the ITS sequencing libraries from the sequencing analysis. Shannon and Simpson indices are used to assess the community diversity, while Chao and Ace are used to evaluate the community richness*.

**Figure 3:**
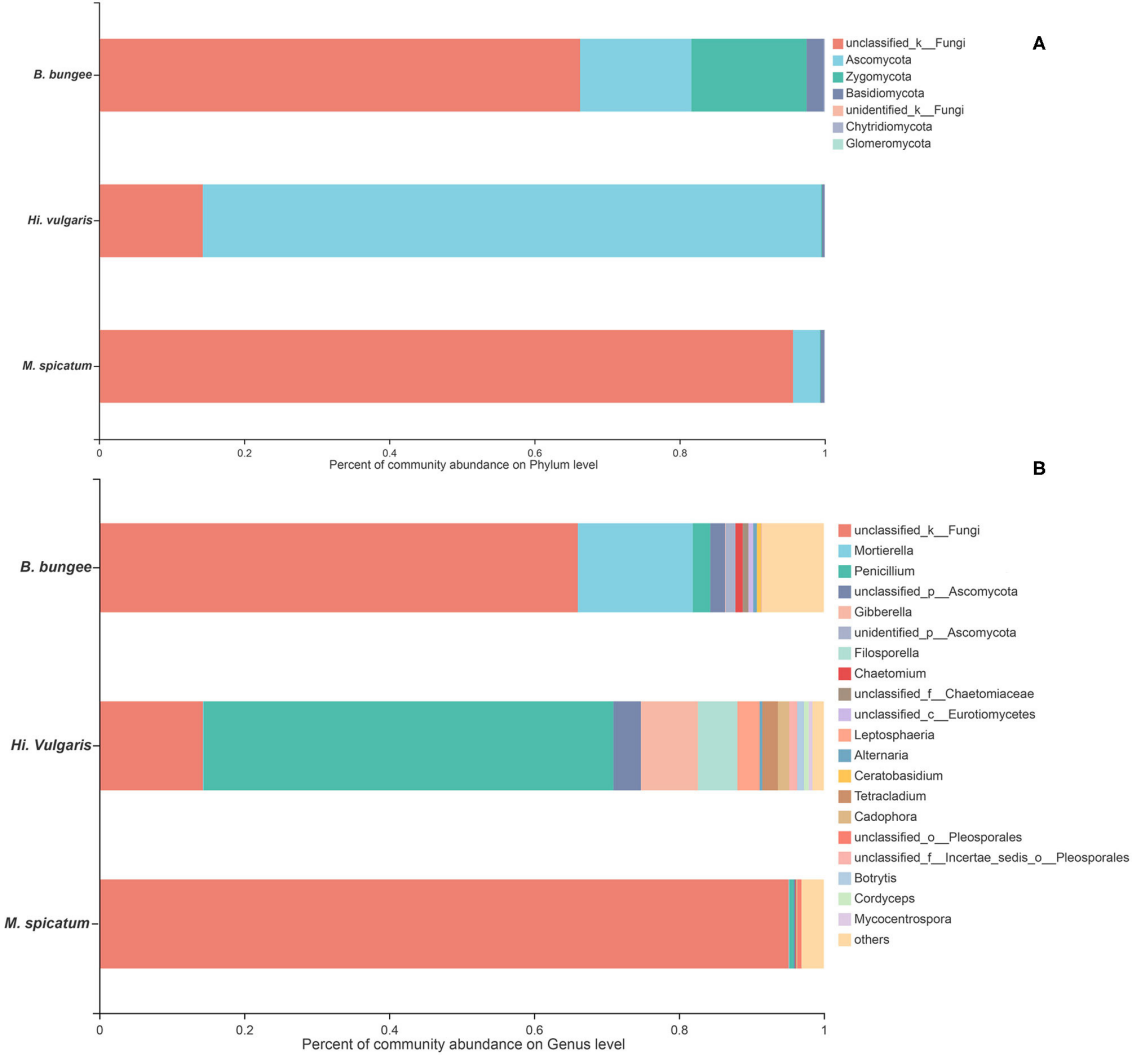
Relative abundance of endophytic fungi in three aquatic plants collected from sampling site LTII at the phylum level **(A)** and genus level **(B)**. When the relative abundance was <0.005%, they were merged together into the “others” group.

In this study, the 1,074 OTUs obtained from all samples were classified into the following six known fungal phyla: Ascomycota (43.48%), Basidiomycota (15.36%), Zygomycota (1.49%), Chytridiomycota (1.21%), Glomeromycota (0.02%), Rozellomycota (0.01%), and several unknown fungal phyla (38.17%). As other studies have reported, Ascomycota is the predominant phylum in aquatic plants (Li et al., 2010; Kohout et al., 2012; Sandberg et al., 2014; You et al., 2015). The relative abundances of the fungal community in three plants at the phylum and genus levels are shown in Figure 3. At the phylum level (Figure 3A), Ascomycota was the dominant phylum in three plants, followed by Basidiomycota in the two plants *H. vulgaris* and *M. spicatum* but Zygomycota in the plant *B. bungei*. At the genus level (Figure 3B), the relatively abundant genus was *Penicillium* in *H. vulgaris* and *M. spicatum* while *Mortierella* in *B. bungei* except unclassified OTUs.

**Figure 4:**
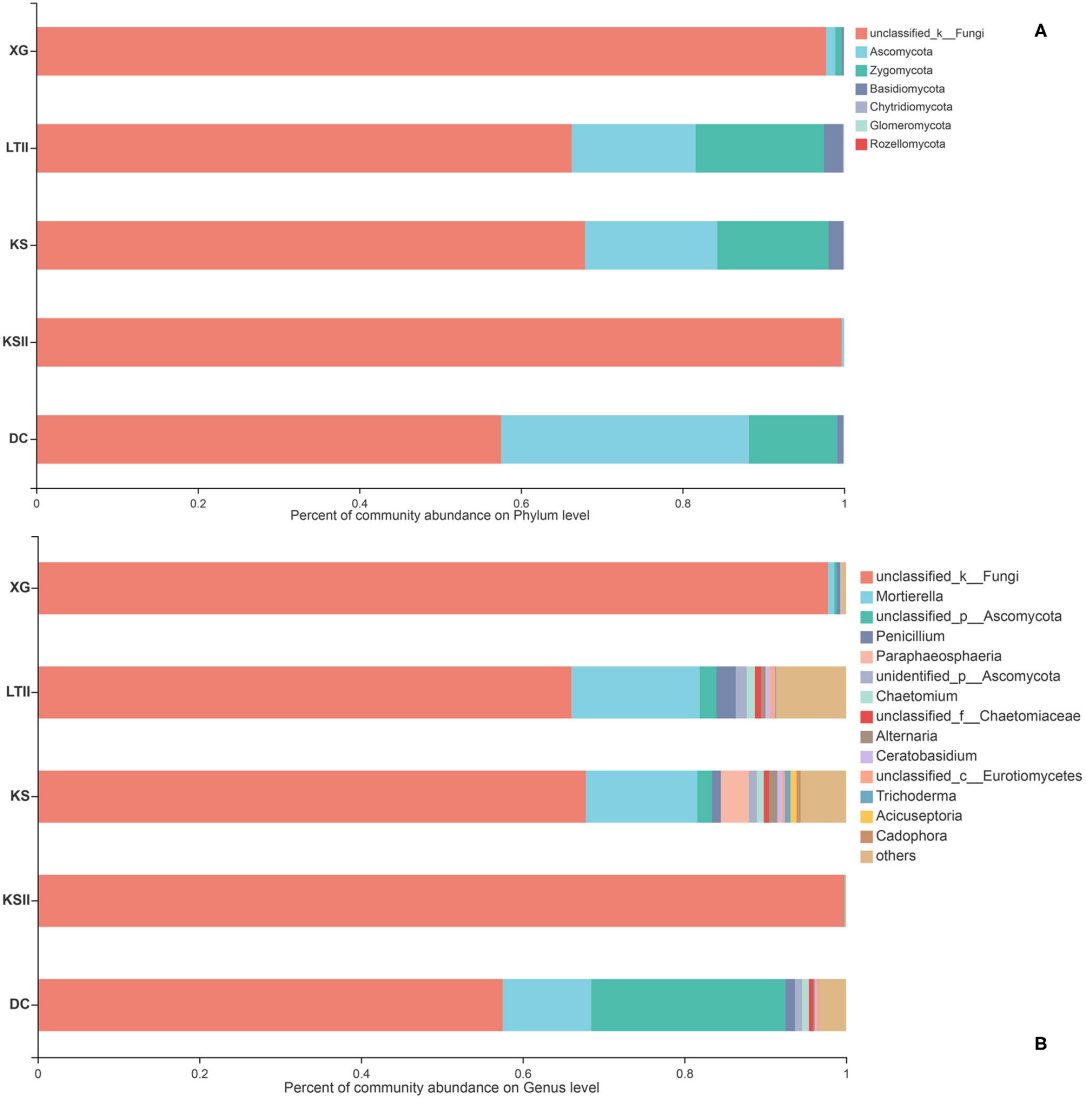
Relative abundance of endophytic fungi of *Batrachium bungei* collected from five sampling sites at the phylum level **(A)** and genus level **(B)**. When the relative abundance was <0.005%, they were merged together into the “others” group.

The relative abundances of fungal community of *B. bungei* among the five sampling sites at the phylum and genus levels are shown in Figure 4. At the phylum level (Figure 4A), the unclassified fungi were the most abundant in sites KSII and XG. Ascomycota was overall the second most common phylum in all samples, followed by Zygomycota. However, Rozellomycota was only found in site KS and Glomeromycota was found in sites LTII, KS, and DC. At the genus level (Figure 4B), *Mortierella* and *Penicillium* were relatively abundant in all samples except unclassified fungi.

**Figure 5:**
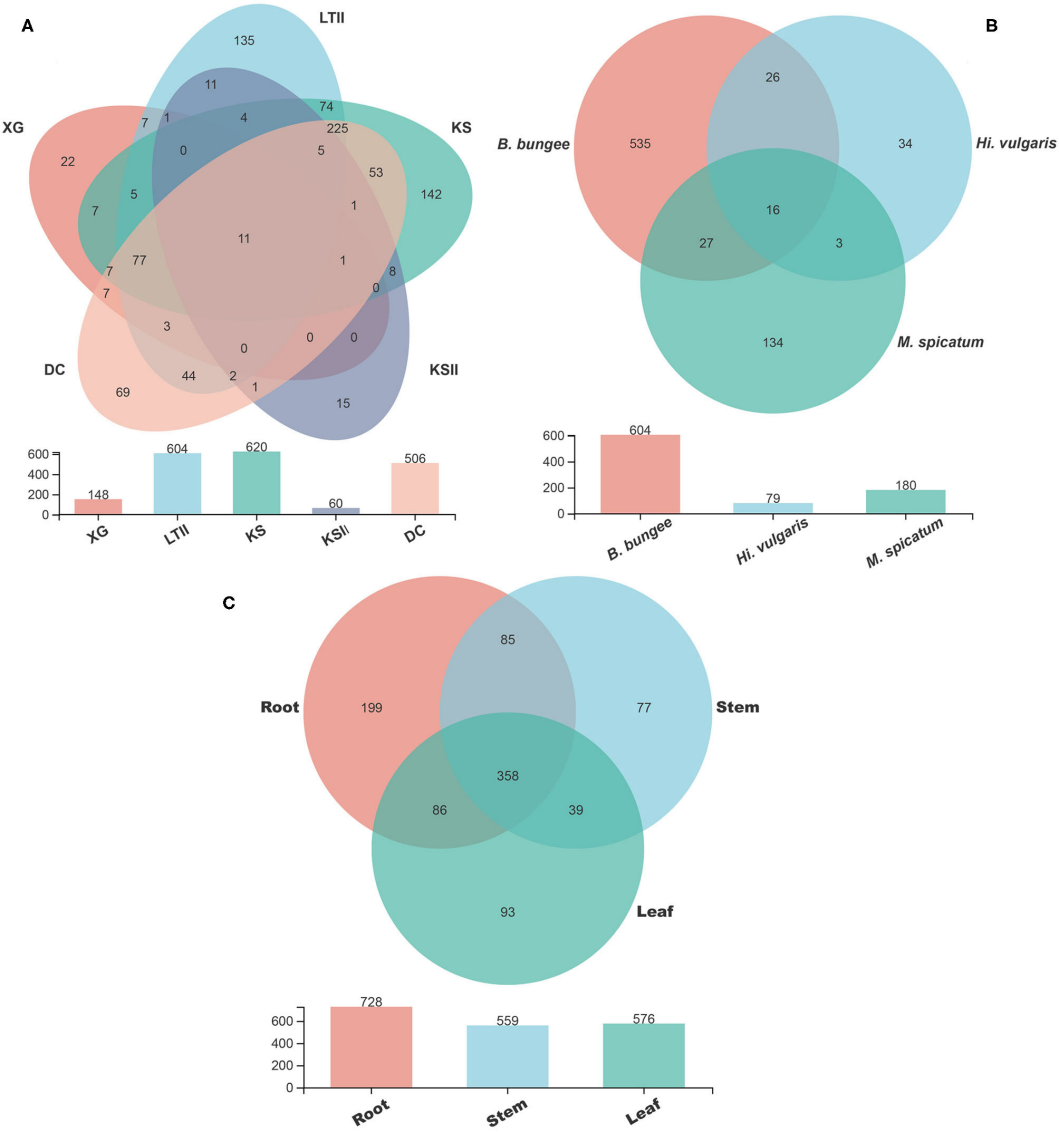
Venn diagram showing the number of fungal OTUs detected in aquatic plants. **(A)** display the number of fungal OTUs of *Batrachium bungei* collected from five sampling sites; **(B)** display the number of fungal OTUs of three different aquatic plants collected from sampling site LTII; **(C)** displays the number of fungal OTUs of different tissues of *B. bungei*. Numbers in the overlapping region indicate shared OTUs between and among the groups. Numbers in the non-overlapping regions indicates unique OTUs for the group.

The Venn diagrams showing the distribution of shared and unique fungal OTUs in the different sampling sites, aquatic plants and plant parts are summarized in Figure 5. Among the five sampling sites, a total of 937 OTUs were obtained from all samples of *B. bungei* (Figure 5A), and 148, 604, 620, 60 and 506 OTUs were obtained from sampling sites XG, LTII, KS, KSII and DC, respectively. There were only 11 OTUs shared among *B. bungei* plants from the five sampling sites, representing 1.2% of the total OTUs, indicating a very low similarity of fungal diversity among different sampling sites. For different aquatic plants in the sampling site LTII, a total of 775 OTUs were discovered among the three plant species, and 180, 79 and 604 OTUs were obtained from plants *M. spicatum, H. vulgaris* and *B. bungei*, respectively. Among these, only 2.1% (16) were shared OTUs (Figure 5B). In contrast, there were 134, 34 and 535 unique OTUs in species *M. spicatum, H. vulgaris* and *B. bungei* respectively. These results revealed that the highest fungal endophyte diversity was in *B. bungei*. Among the three tissue types of *B. bungei* (Figure 5C), there were 728, 559 and 576 OTUs in the root, stem and leaf respectively, and 358 of these OTUs (representing 38.2%) were shared among the three tissue types. The results indicate greater sharing among different tissue types of the same plant than among different plants for the same tissue types.

### Functional Guilds Analysis

**Figure 6:**
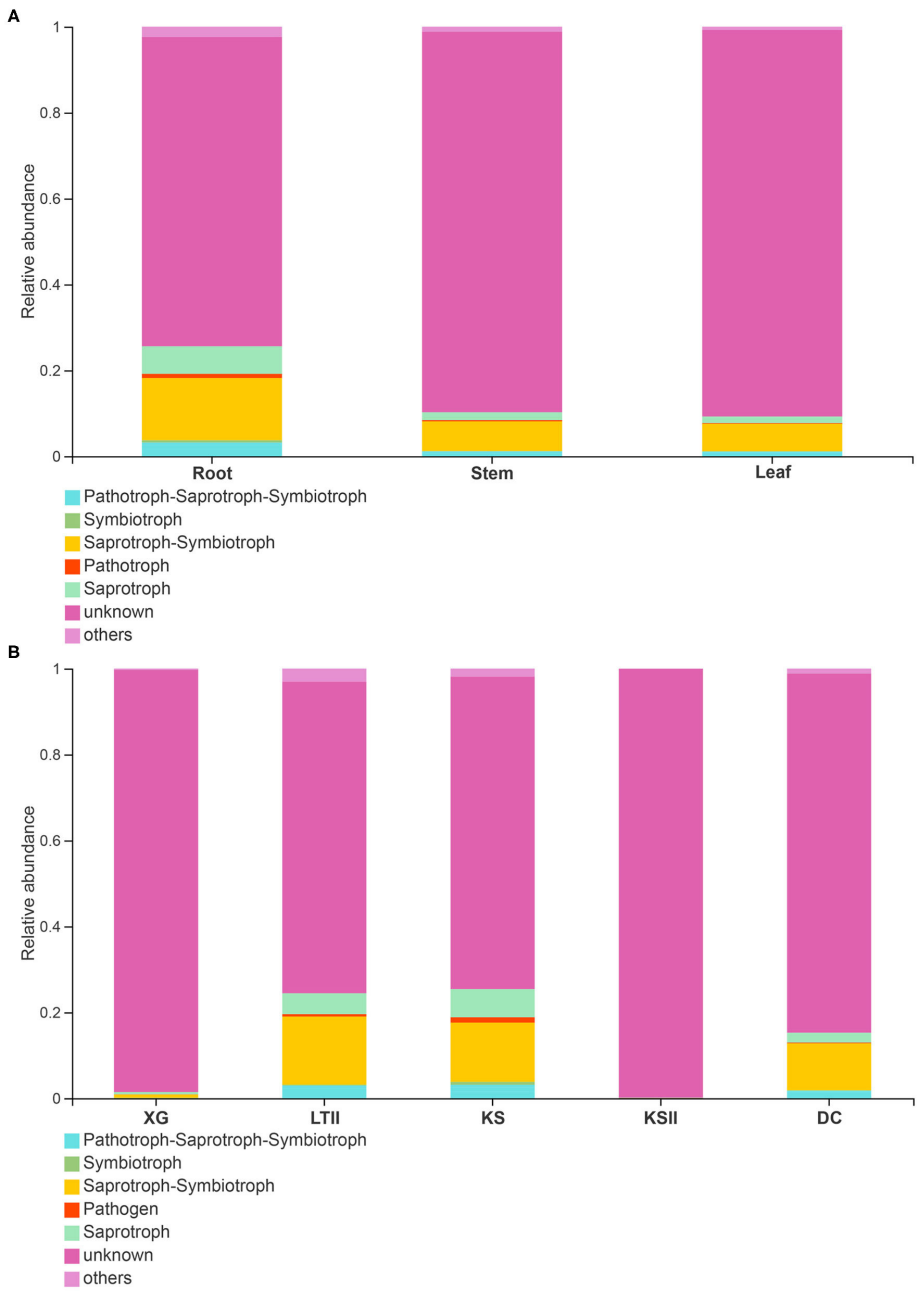
Relative abundance of fungal functional groups (guilds) based on OTUs. **(A)** display the relative abundance in different tissues of *Batrachium bungei*. **(B)** display the relative abundance of *B. bungei* in different sampling sites. When the relative abundance <0.005% will merges into others.

FUNGuild database (http://www.funguild.org/) was used to classify and analyze the ecological guild of endophytic fungi detected in the present study. There are 937 OTUs obtained from the 15 samples of *B. bungei*, which were classified into eight ecological function groups according to the trophic mode (Supplementary Table 5). The unknowns were the most common at 55.39%, followed by members of saprotrophs which represented 19.68% of the total OTUs, pathotroph was the third at 6.52%, pathotroph-saprotroph-symbiotroph was the fourth at 5.99%, symbiotroph was the fifth at 4.71%, saprotroph-symbiotroph and pathotroph-saprotroph were the sixth at 3.10%, and the least was pathotroph-symbiotroph and pathogen-saprotroph-symbiotroph occupying 1.18% and 0.43%, respectively. Among the different tissues of *B. bungei* (Figure 6A), besides unknown functional groups based on OTUs, the relative abundance of saprotroph-symbiotroph was the most abundant in root and stem, but saprotroph was the most abundant in leaf. Among different sampling sites (Figure 6B), besides unknown functional groups, the relative abundance of saprotroph-symbiotroph was the most abundant at four sites, XG, LTII, KS, and DC, but saprotroph was the most abundant in site KSII.

### Fungal Culturing vs. Illumina Sequencing

Culture-based and culture-independent methods were used in this study to estimate endophytic fungal abundance and diversity in aquatic plants in southwestern China. However, Chytridiomycota, Glomeromycota and Rozellomycota were not recovered using culture method. At genus level, a total of 66 genera were detected by two methods. The culture-based method showed that the highest fungal isolation frequency in *B. bungei* was in stem (10.1%), followed by root (8.7%) and leaf (6.5%), whereas the result by Illumina sequencing methods indicated the most abundant OTUs (724) was detected in root, followed by stem (572) and leaf (571). Besides, there were many unclassified or unidentified fungi detected by Illumina sequencing, which likely represent entirely new groups of fungal taxa. Overall, culture-independent method Illumina sequencing detected more species compared to our culture-based method.

## Discussion

The diversity and function of endophytic fungi in terrestrial plants have received substantial attention (Carroll, 1988; Faeth and Hammon, 1997; Higgins et al., 2007; Rodriguez et al., 2009; Suryanarayanan et al., 2011; Ibrahim et al., 2017; Chen et al., 2020). By contrast, the fungal communities from marine and fresh waters have been relatively neglected. In addition, previous studies on endophytic fungi in aquatic plants have mainly used culture-dependent methods (Li et al., 2010; Sandberg et al., 2014), but the application of next generation sequencing technology to examine microbial populations has revealed a much greater diversity of fungi (Kohout et al., 2012). In this study, we studied endophytic fungal diversity in aquatic plants from Southwest China by using both culture-dependent and culture-independent approaches.

Previous studies using cultivation-based methods have investigated the fungal communities in several aquatic plants (Neubert et al., 2006; Arnold and Lutzoni, 2007; Kandalepas et al., 2010; Higgins et al., 2011; Kandalepas, 2012; Kohout et al., 2012; Sandberg et al., 2014; You et al., 2015; Basha et al., 2019), and these studies have revealed a high diversity of endophytes in aquatic plants. Similarly, the fungal diversity we observed using culture-dependent approach in this study was also high (H′ = 5.23, Evenness = 0.72). However, under similar climatic conditions, terrestrial plants such as *Nicotiana tabacum* from Dali (Li et al., 2013), *Dendrobium officinale* from Xishuangbanna (Hu et al., 2010) and nine terrestrial plants from Dawei Mountain (Gao et al., 2019) (all in southwest China), all had high isolation frequencies of endophytic fungi, but with low diversities. Studies in other countries which have similar climatic conditions as southwest China, such as the study on *Kigelia pinnata* from India (Maheswari and Rajagopal, 2013) and *Glycine max* from Brazil (Fernandes et al., 2015), also showed similar results as those in terrestrial plants in southwest China. Together, our study indicates that these aquatic plants in southwest China has relatively high diversity of endophytic fungi, and the high diversity may be related to aquatic environments under similar climatic conditions.

The mean isolation frequency of fungal culture from plant tissues we observed was 11 % among all aquatic plants, and the frequencies did not differ among three different tissue types, with root at 10.7 %, stem at 10.5 % and leaf at 11.5 %. Kohout et al. (2012) reported an isolation frequency of 8.8 % from the roots of five isoetid species from freshwater oligotrophic lakes in southern and central Norway. Sandberg et al. (2014) observed a lower isolation frequency of 2.4 % from three aquatic plants from lakes and reservoirs in Arizona, USA. Therefore, the overall isolation frequency in this study is slightly higher than those reported in previous studies for aquatic plants. In general, the isolation frequency of endophytic fungi in aquatic plants was lower than those observed in terrestrial plants (Kumar and Hyde, 2004; Arnold, 2007; Ibrahim et al., 2017; Gao et al., 2019), with the exception of U'Ren et al. (2010) who recorded a relatively low isolation frequency (7.7 %) in ten terrestrial plants from Arizona.

In terrestrial plants, the high isolation frequency was by Suryanarayanan et al. (2005) who found that the isolation frequency of endophytic fungi in 21 species of cacti was 80%. They reasoned that such a high rate was likely related to the high-water content and long survival period of cacti species. In our study, all plants have high-water content. However, those with long survival period and relatively full-grown tissues, such as *E. crassipes, A. philoxeroides* and *H. dubia*, also had relatively high isolation frequencies, reaching 17.1%, 19.1% and 22.3%, respectively. Yet it is worth noting that even if related studies reported that the isolation frequency of terrestrial plants was positively correlated with rainfall (Wilson and Carroll, 1994; Bills, 1996; Suryanarayanan et al., 1998; Suryanarayanan and Thennarasan, 2004), most research for aquatic plants did not show high abundance of endophytic fungi (Kohout et al., 2012; Sandberg et al., 2014).

Currently, there are few studies using culture-independent techniques to investigate the diversity of endophytic fungi in aquatic plants. In previous study, Kohout et al. (2012) found that roots of aquatic plants have diverse spectrum of mycorrhizal and nonmycorrhizal fungi using culture-dependent and culture-independent techniques. In this study, high-throughput sequencing results revealed a much greater diversity of fungi in aquatic plants than culture-dependent method, and six fungal phyla were detected, including Ascomycota, Basidiomycot, Zygomycot, Chytridiomycota, Glomeromycota, and Rozellomycota. In most studies of fungal diversity in terrestrial plants and aquatic plants, Ascomycota and Basidiomycota were the dominant phylum (Sandberg et al., 2014; You et al., 2015; Al-Bulushi et al., 2017; Ibrahim et al., 2017; Chen et al., 2020). Consistent with previous researches, our study found that fungal communities of *M. spicatum* and *H. vulgaris* are dominated by Ascomycota detected by both culture-independent techniques and culture-dependent method, followed by Basidiomycota and Chytridiomycota. However, culture-independent result of *B. bungei* detected that Ascomycota and Chytridiomycota were the most frequent phyla, followed by Basidiomycota, which this result is similar to the study by Rossmann et al. (2020). Moreover, Huang et al. (2015) also obtained similar result by culture-dependent method.

Comparing the three aquatic plants *B. bungei, H. vulgaris* and *M. spicatum* collected from the same sampling site, the high-throughput sequencing results showed that *B. bungei* had the highest diversity of endophytic fungi, followed by *M. spicatum* and *Hi. Vulgaris* (Figure 5B). However, using culture-based method to analyze the isolation frequency and diversity of endophytic fungi in different aquatic plants, these indexes indicated *M. spicatum* had the highest diversity (H′ = 5.4, Evenness = 0.87) and *B. bungei* had the lowest diversity (H′ = 3.78, Evenness = 0.79). Considering the result difference between two methods, we speculate that the surface disinfection treatment might have a greater impact on *B. bungei* than on *M. spicatum* and *Hi. vulgaris*. Previous studies have reported that the surface sterilization method of plant materials has a great influence on the growth of endophytes (Schulz et al., 1993; Lodge et al., 1996; Arnold et al., 2000; Kohout et al., 2012). Kohout et al. (2012) suspected that bleaches such as sodium hypochlorite might have infiltrated plant epidermis and damaged endophytic fungal structures.

It is known and accepted that the fungal communities are not randomly assembled (Bulgarelli et al., 2012), and previous studies have demonstrated the role played by the plant recruiting a particular microbial consortium to adapt itself to the environmental conditions (Lê Van et al., 2017), including fungal endophyte communities (D'Amico et al., 2018; Illescas et al., 2020). Comparing the three aquatic plants collected from the same sampling site, only 2.1% (16) were shared OTUs, and more OTUs were unique for each plant. However, for different tissues of *B. bungei*, there are more shared OTUs among root, stem, and leaf, occupying 38.2% (358). Therefore, these observations are in agreement with previous reports, which indicate the major role of plant species in shaping the fungal communities (Vandenkoornhuyse et al., 2015; D'Amico et al., 2018; Rossmann et al., 2020).

Using the FUNGuild database to analyze the ecological guilds of endophytic fungi in *B. bungei*, besides the unknown functional groups, most of the fungal OTUs in this study were identified as saprotroph-symbiotroph, followed by symbiotroph and pathotroph-saprotroph. Some of these OTUs were classified as pathotroph, indicating their potential as plant pathogens. As shown in Figure 5, the abundance of pathotrophs was highest in root tissues of *B. bungei*, followed by stem and leaf. Among the five sampling sites, the abundance of pathotroph was highest in sites KS and LTII. What is noteworthy is that the two sites KS and LTII have more human activities compared with the other three sites.

In general, high-throughput sequencing technologies could detect more fungi than the culture-based method in investigation of fungal species diversity. Sugiyama et al. (2010) estimated approximately 1% of the total microbes could be cultured. Even so, some fungi can easily be cultured even when they are present in small quantities. Currently, the detection of the precise diversity of fungi using culture-based techniques is still challenging. In this study, the results also indicate that a higher fungal diversity in aquatic plants was found using high-throughput sequencing than a culture-dependent approach. Moreover, there are many unclassified or unidentified fungi detected by Illumina sequencing in aquatic plants from southwest China, implying that new microbial resources present in this area.

This study enlarges our knowledge of the fungal community of aquatic plants in southwest China. However, there are some limitations presenting in study. For the Illumina sequencing approach, including DNA extractions, preparation of PCR libraries and sequencing, three plant samples were divided into root, stem, and leaf for sequencing respectively, but are in absence of biological triplicates. In fact, several previous studies have shown the high variability among biological replicates (Al-Bulushi et al., 2017; Illescas et al., 2020), although they were performed in fungal endophytes from terrestrial systems, included crop plants (Chen et al., 2018). In addition, Tibet, which also located in southwest China, has a special geographical climate, and the investigation of this area could better understand fungal diversity in southwest China. Further studies should investigate fungal community of aquatic plants in more plant species and sampling sites by high-throughput sequencing technologies in this aera. The application of metagenome sequencing could further help identify the fungal species and their functions to help reduce the existing gap between culture-independent and culture-dependent method. Notably, using different media may help uncover different groups of culturable fungi (Li et al., 2010; Kohout et al., 2012), which could be used in future studies of aquatic fungi. In addition to fungi, there are many bacteria associated with aquatic plants (Chen et al., 2012; Rybakova and Kopylov, 2017). More studies need to be conducted in this topic in the future.

## Conclusion

To conclude, our study reveals that aquatic plants in Southwest China contains abundant endophytic fungi diversity. We investigated the diversity of endophytic fungi in 30 aquatic plants by fungal culturing. The result showed that the average of isolation frequency of all plants is 11%, and the average of isolation frequency did not differ significantly among the three tissue types. Although isolation frequency was relatively low, endophytes in aquatic plants collected from Southwest China were diverse and species-rich (H′ = 5.23, Evenness = 0.72). In addition, as altitude increased, the isolation frequency, richness of fungal species and diversity index of endophytic fungi tended to decrease. Further research on the three most common plants using culture-independent technique showed that *B. bungei* has the most abundant OTUs. Among three tissue types of *B. bungei*, both the number of total OTUs and unique OTUs in root are higher than those in stem and leaf. Our study expands our knowledge in endophytic fungi diversity in aquatic plants in southwest China. Notably, there were a large number of unclassified and unidentified fungi detected by Illumina sequencing. These fungi represent potential resources for further exploration.

## Data Availability Statement

The original contributions presented in the study are included in the article/Supplementary Material, further inquiries can be directed to the corresponding author.

## Author Contributions

ZY conceived and designed the study. HZ wrote the manuscript. MQ and HZ conducted the experiments and analyzed the data. ZY and JX revised the manuscript. All authors read and approved the final manuscript.

## Conflict of Interest

The authors declare that the research was conducted in the absence of any commercial or financial relationships that could be construed as a potential conflict of interest.

## Publisher's Note

All claims expressed in this article are solely those of the authors and do not necessarily represent those of their affiliated organizations, or those of the publisher, the editors and the reviewers. Any product that may be evaluated in this article, or claim that may be made by its manufacturer, is not guaranteed or endorsed by the publisher.
