# Supplementary material for: Culture-Based and Culture-Independent Assessments of Endophytic Fungal Diversity in Aquatic Plants in Southwest China
Source: Front Fungal Biol. 2021 Jul 27;2:692549. doi: 10.3389/ffunb.2021.692549 (PMC10512276; doi:10.3389/ffunb.2021.692549)

**Supplementary Figure S1:** Rarefaction curve analysis of all samples based on the Sobs index of OUT level.


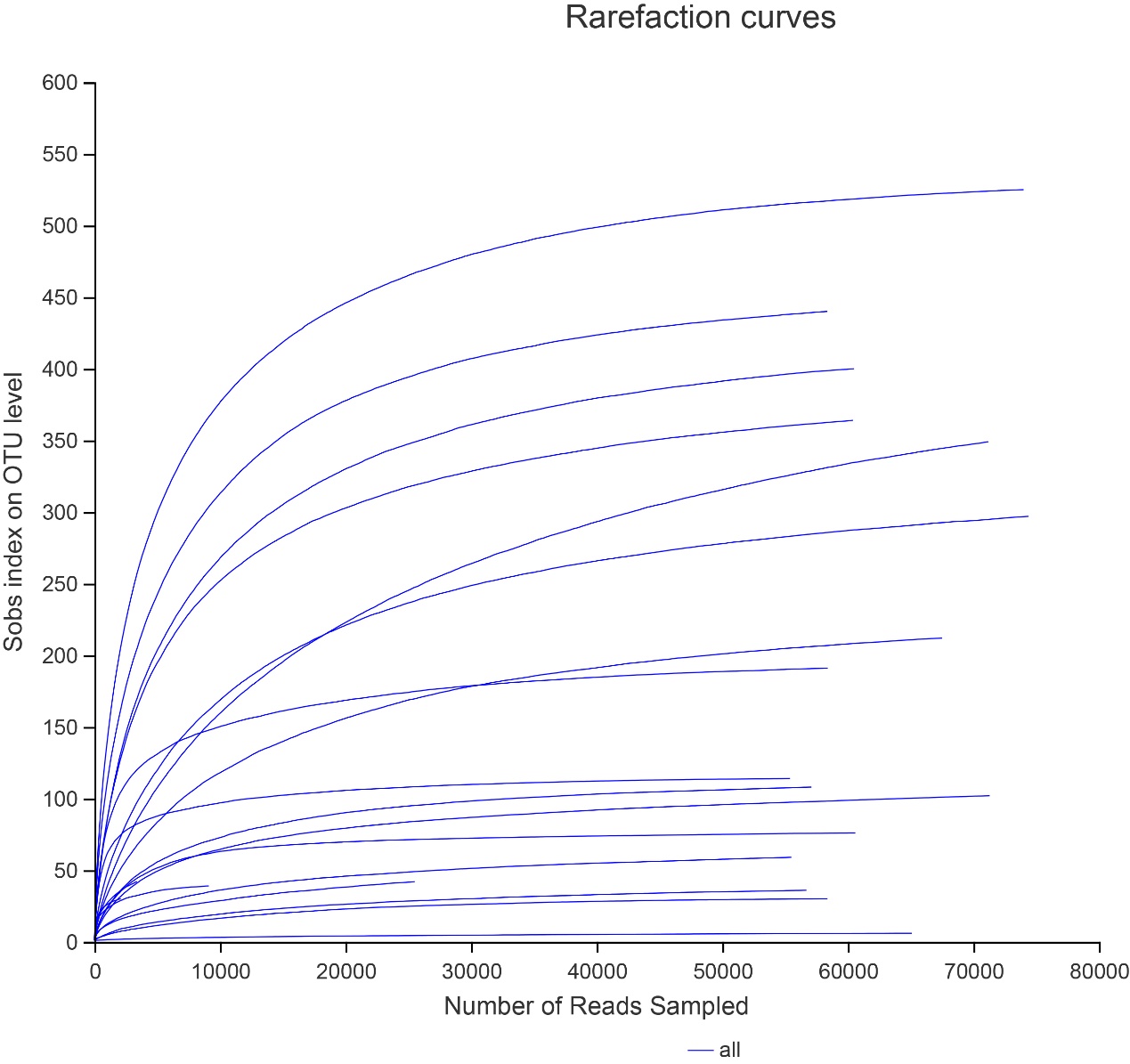

Supplement: Supplementary file 1 [file Data_Sheet_1.docx]
