# Supplementary material for: Culture-Based and Culture-Independent Assessments of Endophytic Fungal Diversity in Aquatic Plants in Southwest China
Source: Front Fungal Biol. 2021 Jul 27;2:692549. doi: 10.3389/ffunb.2021.692549 (PMC10512276; doi:10.3389/ffunb.2021.692549)
Supplement: Supplementary file 2 [file Table_1.docx]

**TABLE S1.** Locations and characteristics of sampling sites for surveys of endophytic fungi in aquatic plants in Southwest China.

| **Province** | **District** | **Code** | **Microsite** | **Latitude** | **Longitude** | **Elevation (m)** | **Type** |
| --- | --- | --- | --- | --- | --- | --- | --- |
| **Yunnan** | Dianchi | D | 21 | E102°45'19.39" | N24°47'10.18" | 1895 | Lake |
|  | Erhai | E | 20 | E100°13'15.03" | N25°51'10.05" | 1979 | Lake |
|  | Fuxianhu | F | 12 | E102°56'26.59" | N24°31'00.55" | 1727 | Lake |
|  | Jianhu | JH | 5 | E099°46'21.56" | N26°30'21.02" | 2190 | Lake |
|  | Lashihai | L | 3 | E100°08'48.72" | N26°53'58.93" | 2455 | Lake |
|  | Wenbihai | WB | 1 | E100°12'48.47" | N26°49'20.20" | 2431 | Lake |
|  | Heilongtan | LH | 1 | E100°13'56.17" | N26°52'50.86" | 2415 | River |
|  | Huamajie | HM | 1 | E100°12'25.09" | N26°53'20.18" | 2420 | River |
|  | Yangzonghai | Y | 8 | E103°01'32.37" | N24°57'31.86" | 1770 | Lake |
|  | Tengchong | T | 6 | E098°34'58.86" | N25°00'11.93" | 2063 | Pond |
|  | Beimiao | BM | 1 | E099°12'43.52" | N25°14'12.47" | 2438 | Reservoir |
|  | Banqiao | BB | 1 | E099°13'16.84" | N25°12'20.60" | 1668 | River |
|  | Xianggelila | X | 20 | E099°44'02.29" | N27°52'56.75" | 3305 | Lake |
|  | Yila | YL | 4 | E099°39'33.73" | N27°52'16.80" | 3269 | Wetland |
|  | Gongkahu | G | 1 | E98°90'99.12" | N28°49'77.36" | 3400 | Lake |
|  | Xiajisha | XG | 2 | E099°49'02.06" | N27°27'49.28" | 3275 | Wetland |
| **Guizhou** | Chaohai | CH | 1 | E104°23'01.31" | N26°84'68.44" | 2200 | Lake |
|  | Huaxi | HX | 2 | E106°40'27.15" | N26°26'31.96" | 1081 | Wetland |
|  | Hongfenghu | HF | 1 | E106°23'57.04" | N26°29'59.71" | 1243 | Lake |
|  | Ahahu | AH | 1 | E106°39'42.77" | N26°32'40.80" | 1064 | Lake |
| **Sichuan** | Qionghai | QH | 1 | E102°28'02.98" | N27°83'77.36" | 1520 | Lake |
|  | Luding | LD | 1 | E102°15'08.3" | N29°45'57.4" | 2077 | Pond |
|  | Ruogaier | RH | 1 | E102°49'02.2" | N38°55'35" | 3434 | Lake |
|  | Litang | LT | 2 | E100°07'05.1" | N30°04'12.4" | 4029 | Pond |
|  | Qisehai | QS | 1 | E101°52'16.6" | N30°11'0" | 3262 | Lake |
|  | Wuxuhai | WX | 1 | E101°23'56.7" | N29°09'35.8" | 3708 | Lake |
|  | Daofu | DF | 1 | E101°06'04.9" | N30°58'49.7" | 3266 | Pond |
|  | Kashahu | KS | 2 | E100°15'31.9" | N31°39'07.5" | 3505 | Lake |
|  | Daocheng | DC | 1 | E100°14'46.5" | N29°29'48.5" | 4362 | Pond |
|  | Baiyu | BY | 1 | E99°41'11.1" | N31°37'30.7" | 4013 | Pond |
|  | Ganzi | GZ | 2 | E100°13'22.6" | N31°06'12.3" | 4198 | Pond |

**Notes:** Latitude, longitude, and elevation indicate central sampling location among multiple microsites per reservoir.

**TABLE S2.** The GenBank accession numbers of partly isolates generated in this study.

| **Taxa** | Isolates | Accession No. | **Taxa** | Isolates | Accession No. |
| --- | --- | --- | --- | --- | --- |
| *Acremonium* sp. | D135 | MZ380012 | *Myrothecium* sp*.* | L23 | MZ380090 |
| *Acrostalagmus* sp. | F86 | MZ380013 | *Nemania* sp. | E46 | MZ380091 |
| *Alternaria* sp. | D10 | MZ380014 | *Neoascochyta* sp. | DF23 | MZ380092 |
| *Ampelomyces* sp. | D48 | MZ380015 | *Neofabraea* sp. | BY52 | MZ380093 |
| *Annulohypoxylon* sp. | DF43 | MZ380016 | *Neurospora* sp. | JH7 | MZ380094 |
| *Apodus* sp. | E77 | MZ380017 | *Nigrospora* sp. | D84 | MZ380095 |
| *Arthrinium* sp. | QS24 | MZ380018 | *Oedocephalum* sp. | G30 | MZ380096 |
| *Articulospora* sp. | G67 | MZ380019 | *Oliveonia* sp. | LH2 | MZ380097 |
| *Aspergillus* sp. | E22 | MZ380020 | *Orbilia* sp. | D27 | MZ380098 |
| *Athelia* sp. | E30 | MZ380021 | *Paraconiothyrium* sp. | F82 | MZ380099 |
| *Aureobasidium* sp. | KS79 | MZ380022 | *Penicillium* sp. | E74 | MZ380100 |
| *Beauveria* sp. | E130 | MZ380023 | *Peniophora* sp. | E110 | MZ380101 |
| *Biscogniauxia* sp. | E104 | MZ380024 | *Perenniporia* sp. | HF8 | MZ380102 |
| *Bjerkandera* sp. | D21 | MZ380025 | *Periconia* sp. | X9 | MZ380103 |
| *Boeremia* sp. | DF33 | MZ380026 | *Pestalotiopsis* sp. | LH17 | MZ380104 |
| *Botryosphaeria* sp. | D4 | MZ380027 | *Peyronellaea* sp. | D131 | MZ380105 |
| *Botrytis* sp. | GZ45 | MZ380028 | *Peziza* sp. | L30 | MZ380106 |
| *Calonectria* sp. | D138 | MZ380029 | *Phaeocytostroma* sp. | JH34 | MZ380107 |
| *Ceratobasidium* sp. | XG81 | MZ380030 | *Phaeophlebiopsis* sp. | QS20 | MZ380108 |
| *Ceratophoma* sp. | D40 | MZ380031 | *Phaeosphaeria* sp. | DF11 | MZ380109 |
| *Cercospora* sp. | E57 | MZ380032 | *Phanerochaete* sp. | E7 | MZ380110 |
| *Chaetomium* sp. | D16 | MZ380033 | *Phialemoniopsis* sp. | DC25 | MZ380111 |
| *Chromelosporium* sp. | E55 | MZ380034 | *Phlebia* sp. | KS66 | MZ380112 |
| *Ciboria* sp. | BY55 | MZ380035 | *Phlebiopsis* sp. | CH45 | MZ380113 |
| *Cladorrhinum* sp. | JH26 | MZ380036 | *Pholiota* sp. | DF44 | MZ380114 |
| *Cladosporium* sp. | D92 | MZ380037 | *Phoma* sp. | KS1 | MZ380115 |
| *Clitopilus* sp. | HX17 | MZ380038 | *Phyllosticta* sp. | X43 | MZ380116 |
| *Clonostachys* sp. | Z4 | MZ380039 | *Physarum* sp. | KS48 | MZ380117 |
| *Colletotrichum* sp. | D9 | MZ380040 | *Pilidiella* sp. | D141 | MZ380118 |
| *Coniochaeta* sp. | JH11 | MZ380041 | *Pilidium* sp. | X11 | MZ380119 |
| *Coniothyrium* sp. | G44 | MZ380042 | *Plectosphaerella* sp. | D5 | MZ380120 |
| *Coprinellus* sp. | D25 | MZ380043 | *Pleiochaeta* sp. | DC39 | MZ380121 |
| *Coprinopsis* sp. | D72 | MZ380044 | *Plenodomus* sp. | DC6 | MZ380122 |
| *Cordyceps* sp. | KS23 | MZ380045 | *Podospora* sp. | BY37 | MZ380123 |
| *Corynascella* sp. | BY6 | MZ380046 | *Porostereum* sp. | QH44 | MZ380124 |
| *Cudoniella* sp. | KS78 | MZ380047 | *Preussia* sp. | X8 | MZ380125 |
| *Curvularia* sp. | D55 | MZ380048 | *Psathyrella* sp. | LH1 | MZ380126 |
| *Cylindrocarpon* sp. | F55 | MZ380049 | *Pseudeurotium* sp. | BY38 | MZ380127 |
| *Cylindrocladiella* sp. | D122 | MZ380050 | *Psilocybe* sp. | QH62 | MZ380128 |
| *Cytosporina* sp. | D15 | MZ380051 | *Pyrenochaeta* sp. | L12 | MZ380129 |
| *Dactylonectria* sp. | E76 | MZ380052 | *Pyrenochaetopsis* sp. | L10 | MZ380130 |
| *Daldinia* sp. | HF11 | MZ380053 | *Pyronema* sp. | GZ65 | MZ380131 |
| *Diaporthe* sp. | D14 | MZ380054 | *Pythium* sp. | D54 | MZ380132 |
| *Didymella* sp. | E65 | MZ380055 | *Rhizoctonia* sp. | XG65 | MZ380133 |
| *Dimorphospora* sp. | X46 | MZ380056 | *Rhizopus* sp. | E98 | MZ380134 |
| *Dokmaia* sp. | L29 | MZ380057 | *Rosellinia* sp. | DF13 | MZ380135 |
| *Echinodontium* sp. | WX6 | MZ380058 | *Saccharicola* sp. | LT28 | MZ380136 |
| *Entoleuca* sp. | LT51 | MZ380059 | *Sarocladium* sp. | HF2 | MZ380137 |
| *Epicoccum* sp. | WX5 | MZ380060 | *Scedosporium* sp. | E64 | MZ380138 |
| *Eutypella* sp. | LT36 | MZ380061 | *Schizophyllum* sp. | E53 | MZ380139 |
| *Filosporella* sp. | X87 | MZ380062 | *Schizothecium* sp. | KS50 | MZ380140 |
| *Fimetariella* sp. | JH17 | MZ380063 | *Sclerotinia* sp. | F42 | MZ380141 |
| *Fusarium* sp. | D47 | MZ380064 | *Septoria* sp. | GZ56 | MZ380142 |
| *Ganoderma* sp. | E107 | MZ380065 | *Setomelanomma* sp. | BY34 | MZ380143 |
| *Geomyces* sp. | DC26 | MZ380066 | *Setophaeosphaeria* sp. | QH34 | MZ380144 |
| *Geotrichum* sp. | YL1 | MZ380067 | *Sigmoidea* sp. | D97 | MZ380145 |
| *Heterobasidion* sp. | QS12 | MZ380068 | *Sistotrema* sp. | QH56 | MZ380146 |
| *Heteroconium* sp. | LT43 | MZ380069 | *Sordaria* sp. | X21 | MZ380147 |
| *Heydenia* sp. | LD15 | MZ380070 | *Spegazzinia* sp. | HX8 | MZ380148 |
| *Hirsutella* sp. | G68 | MZ380071 | *Sporormia* sp. | D136 | MZ380149 |
| *Hyphodontia* sp. | E6 | MZ380072 | *Stachybotrys* sp. | HF10 | MZ380150 |
| *Hypholoma* sp. | DF37 | MZ380073 | *Stagonospora* sp. | D87 | MZ380151 |
| *Hypoxylon* sp. | CH37 | MZ380074 | *Stagonosporopsis* sp. | F28 | MZ380152 |
| *Ilyonectria* sp. | D8 | MZ380075 | *Stemphylium* sp. | RH38 | MZ380153 |
| *Irpex* sp. | D82 | MZ380076 | *Talaromyces* sp. | Y9 | MZ380154 |
| *Isaria* sp. | F61 | MZ380077 | *Tetracladium* sp. | RH28 | MZ380155 |
| *Lecanicillium* sp. | F35 | MZ380078 | *Thyrostroma* sp. | DC4 | MZ380156 |
| *Leptodontidium* sp. | BY43 | MZ380079 | *Trametes* sp. | E63 | MZ380157 |
| *Leptosphaerulina* sp. | D147 | MZ380080 | *Trichoderma* sp. | D142 | MZ380158 |
| *Libertella* sp. | E33 | MZ380081 | *Trichosporon* sp. | QH81 | MZ380159 |
| *Massarina* sp. | E12 | MZ380082 | *Truncatella* sp. | G46 | MZ380160 |
| *Merimbla* sp. | F80 | MZ380083 | *Varicosporium* sp. | X37 | MZ380161 |
| *Meyerozyma* sp. | DF39 | MZ380084 | *Virgariella* sp. | CH36 | MZ380162 |
| *Microdochium* sp. | E66 | MZ380085 | *Westerdykella* sp. | HX22 | MZ380163 |
| *Microsphaeropsis* sp. | D134 | MZ380086 | *Xylaria* sp. | LH6 | MZ380164 |
| *Monodictys* sp. | X58 | MZ380087 | *Xylocoremium* sp. | JH13 | MZ380165 |
| *Mortierella* sp. | D117 | MZ380088 | *Zopfiella* sp. | GZ31 | MZ380166 |
| *Mycocentrospora* sp. | XG60 | MZ380089 | *Zygosporium* sp. | D101 | MZ380167 |

**TABLE S3.** Taxonomic information of all endophytic fungi isolated from aquatic plants in Southwest China.

| **Genera (asexual/sexual)** | **Family** | **Order** | **Class** | **Phylum** | **Isolates** |
| --- | --- | --- | --- | --- | --- |
| *Alternaria/Lewia* | Pleosporaceae | Pleosporales | Dothideomycetes | Ascomycota | D10,D19,D20,D42,D45,D46,D61,D71,D73,D80,D88,D89,D95,D105,D115,D118,D126,D129,D149,E10,E16,E58,E96,G14,G21,G36,F8,F14,F44,F57,F62,F67,F70,X7,X29,X32,X38,X65,JH10,JH28,JH41,Y14,Y21,CH30,CH40,BY3,BY10,BY27,BY72,HX20,KS24,KS40,KS36,QH14,QH23,QH50,RH15,RH16,GZ34,GZ86,GZ36,GZ26,GZ74,GZ77,GZ84,XG61 |
| *Stemphylium* | Pleosporaceae | Pleosporales | Dothideomycetes | Ascomycota | RH38 |
| *Epicoccum* | Pleosporaceae | Pleosporales | Dothideomycetes | Ascomycota | X55,X56,T2,T3,WX5,WX8 |
| *Sporormia* | Sporormiaceae | Pleosporales | Dothideomycetes | Ascomycota | D136 |
| *Preussia* | Sporormiaceae | Pleosporales | Dothideomycetes | Ascomycota | G41,X8,X77,YL14,YL18,KS41 |
| *Westerdykella* | Sporormiaceae | Pleosporales | Dothideomycetes | Ascomycota | HX22 |
| *Ampelomyces* | Phaeosphaeriaceae | Pleosporales | Dothideomycetes | Ascomycota | D48,F24,HM12 |
| *Stagonospora*/*Phaeosphaeria* | Phaeosphaeriaceae | Pleosporales | Dothideomycetes | Ascomycota | D87,D104,BY76 |
| *Setomelanomma* | Phaeosphaeriaceae | Pleosporales | Dothideomycetes | Ascomycota | BY34,BY63 |
| *Setophaeosphaeria* | Phaeosphaeriaceae | Pleosporales | Dothideomycetes | Ascomycota | HM8,L31,QH34 |
| *Peyronellaea*/*Didymella* | Didymellaceae | Pleosporales | Dothideomycetes | Ascomycota | D131,D113,E43,E48,E124,HM2,HM3,HM4,JH24,L21,CH25,GZ85,DF15 |
| *Stagonosporopsis* | Didymellaceae | Pleosporales | Dothideomycetes | Ascomycota | F28,YL26,GZ60,GZ69,GZ73,GZ76,QH71,LT71,LT72,DF52 |
| *Boeremia* | Didymellaceae | Pleosporales | Dothideomycetes | Ascomycota | DF33 |
| *Neoascochyta* | Didymellaceae | Pleosporales | Dothideomycetes | Ascomycota | DF23 |
| *Microsphaeropsis*/*Paraphaeosphaeria* | Montagnulaceae | Pleosporales | Dothideomycetes | Ascomycota | D78,D134 |
| *Paraconiothyrium*/*Paraphaeosphaeria* | Montagnulaceae | Pleosporales | Dothideomycetes | Ascomycota | F82,X3,X4,X15,X48,X49,X86,L11,L50,ZG8,GZ11,GZ13,KS71,KS11,HX23,LD8,RH39,XG33,XG84,Z3,Z9,Z19,Z20,Z21,ZG3,DF25 |
| *Saccharicolal* | Massarinaceae | Pleosporales | Dothideomycetes | Ascomycota | LT28,LT32,LT53,LT6,LT64,QH64,XG53,XG89 |
| *Ceratophoma* | Massarinaceae | Pleosporales | Dothideomycetes | Ascomycota | D40,D44,D49,D93,D94,D99,D107,E1,E5,E19,E26,E36,E40,E47,E61,E90,E115,E119,E120,E138,E139,G2,G9,G11,G12,G13,G17,G18,G20,G38,G42,G43,G47,G48,G49,G50,G51,G59,G60,G61,G62,G69,G70,G71,G72,G73,G74,G78,F11,F15,F30,F31,F60,F64,F69,F75,JH35,JH36,X16,X18,X33,X40,X41,X71,X74,X78,X81,X83,X89,L1,L8,L16,YL12,YL15,YL20,YL23,YL24,YL25,YL28,Z11,Z16,CH38,CH14,CH17,DF8 |
| *Acrocalymma*/*Massarina* | Lophiostomataceae | Pleosporales | Dothideomycetes | Ascomycota | E12,RH23,XG16,XG26 |
| *Coniothyrium*/*Leptosphaeria* | Leptosphaeriaceae | Pleosporales | Dothideomycetes | Ascomycota | G44,G58,X35,X79,HM12,LH5,GZ51 |
| *Pyrenochaetopsis* | Cucurbitariaceae | Pleosporales | Dothideomycetes | Ascomycota | L10 |
| *Plenodomus* | Incertae sedis | Pleosporales | Dothideomycetes | Ascomycota | E13,X69,DC6,QS31,LT10,XG7,XG54 |
| *Pyrenochaeta*/*Herpotrichia*,*Leptosphaeria* | Incertae sedis | Pleosporales | Dothideomycetes | Ascomycota | D18,L12,L13 |
| *Phoma*/*Didymella* | Incertae sedis | Pleosporales | Dothideomycetes | Ascomycota | D29,D40,D44,D49,D93,D94,D99,D107,E1,E5,E19,E26,E36,E40,E47,E61,E90,E115,E119,E120,E138,E139,G2,G9,G11,G12,G13,G17,G18,G20,G38,G42,G43,G47,G48,G49,G50,G51,G59,G60,G61,G62,G69,G70,G71,G72,G73,G74,G78,F11,F15,F30,F31,F60,F64,F69,F75,JH35,JH36,X16,X18,X33,X40,X41,X71,X74,X78,X81,X83,X89,L1,L8,L16,Y10,Y13,YL12,YL15,YL20,YL23,YL24,YL25,YL28,Z11,Z16,BY105,BY53,BY86,BY84,BY85,BY2,BY54,BY79,BY36,BY42,BY92,BY5,BY89,BY91,BY90,BY78,CH38,CH14,CH17,DF8,DF34,DF57,DC3,KS1,KS4,KS5,KS6,KS15,KS18,KS19,KS20,KS33,KS34,KS43,KS46,KS47,KS52,KS58,KS59,KS75,LD9,LD25,QS40,QS43,RH6,RH13,RH25,XG15,XG27,XG30,XG31,HX19,GZ8,GZ38,GZ39,GZ43,GZ57,GZ58,GZ59,LT15,LT7,LT8,LT16,LT22,LT35,LT37,LT56,LT59,LT65,LT85,LT91,LT95,LT98,LT99,LT107,LT110,LT127,LT128,LT129,LT130,LT131,LT132,LT133,LT134,LT137,LT139,LT149,LT156,LT157,LT161,LT164,LT186,QH86,QH88 |
| *Mycocentrospora* | Incertae sedis | Pleosporales | Dothideomycetes | Ascomycota | XG60,XG68 |
| *Thyrostroma* | Botryosphaeriaceae | Botryosphaeriales | Dothideomycetes | Ascomycota | DC4 |
| *Phyllosticta*/*Guignardia* | Botryosphaeriaceae | Botryosphaeriales | Dothideomycetes | Ascomycota | X43 |
| *Cercospora*/*Mycosphaerella* | Mycosphaerellaceae | Capnodiales | Dothideomycetes | Ascomycota | E57 |
| *Septoria* | Mycosphaerellaceae | Capnodiales | Dothideomycetes | Ascomycota | GZ52,GZ56,KS67 |
| *Cladosporium*/*Davidiella* | Davidiellaceae | Capnodiales | Dothideomycetes | Ascomycota | D92,D140,D154,F49,G52,G53,G65,G81,X5,X30,X52,X57,X62,X63,X82,X91,X92,HM1,JH9,L25,LH15,Y12,BY11,BY25,BY35,DF3,DF42,DF21,LT20,LT100,QH42,KS49,KS61,HX12 |
| *Heteroconium*/*Antennulariella* | Antennulariellaceae | Capnodiales | Dothideomycetes | Ascomycota | LT43,LT45 |
| *Aureobasidium* | Dothioraceae | Dothideales | Dothideomycetes | Ascomycota | KS79,RH27 |
| *Ascochyta*/*Didymella* | Incertaesedis | Incertaesedis | Dothideomycetes | Ascomycota | E27,E65,E92,F10,CH27 |
| *Monodictys* | Incertae sedis | Incertae sedis | Incertae sedis | Ascomycota | X58 |
| *Lecythophora*/*Coniochaeta* | Coniochaetaceae | Coniochaetales | Sordariomycetes | Ascomycota | JH11,JH22,QH57 |
| *Cylindrocladium*/*Calonectria* | Nectriaceae | Hypocreales | Sordariomycetes | Ascomycota | D138,E29,F68,GZ6,HF5,LD37 |
| *Clonostachys*/*Bionectria* | Nectriaceae | Hypocreales | Sordariomycetes | Ascomycota | Z2,Z4,Z8,Z10,Z12,Z14,Z18,Z24,Z25,T11,X25 |
| *Cylindrocarpon*/*Neonectria* | Nectriaceae | Hypocreales | Sordariomycetes | Ascomycota | F55 |
| *Cylindrocladiella*/*Nectricladiella* | Nectriaceae | Hypocreales | Sordariomycetes | Ascomycota | D122 |
| *Dactylonectria* | Nectriaceae | Hypocreales | Sordariomycetes | Ascomycota | E76,X61,X88 |
| *Fusarium*/*Gibberella*,*Haematonectria* | Nectriaceae | Hypocreales | Sordariomycetes | Ascomycota | D47,D50,D53,D62,D63,D67,D103,D106,D124,D143,D145,E3,E68,E75,E102,E118,G1,G8,G10,G16,G19,G29,G34,G37,G39,G45,F7,F27,F32,F40,F41,F51,F53,F66,X23,X27,X54,X73,X76,JH19,JH25,JH29,JH30,JH39,L4,Y3,Y4,Y5,YL8,YL10,YL13,Z1,Z6,Z17,BY22,BY93,BY95,BY103,DC1,DC2,DC5,DC9,DC11,DC12,DC15,DC17,DC18,DC19,DC20,DC21,DC24,DC53,LD3,LD24,HX3,LT11,LT112,LT117,LT12,LT122,LT123,LT125,LT13,LT135,LT138,LT14,LT140,LT150,LT152,LT155,LT159,LT160,LT162,LT163,LT170,LT171,LT172,LT173,LT176,LT178,LT179,LT184,LT191,LT29,LT30,LT38,LT40,LT57,LT81,LT83,LT87,QH10,QH100,QH17,QH29,QH30,QH71,QH73,QH75,QH91,XG1,XG2,XG3,XG4,XG5,XG6,XG8,XG9,XG10,XG13,XG14,XG17,XG18,XG19,XG21,XG23,XG24,XG25,XG28,XG36,XG49,XG55,XG56,XG57,XG58,XG59,XG62,XG63,XG64,XG66,XG70,XG74,XG75,XG76,XG77,XG78,XG80,XG86,XG87,XG90,XG91,XG92,XG111,XG125,XG126,XG127 |
| *Acremonium* | Nectriaceae | Hypocreales | Sordariomycetes | Ascomycota | D22,D56,D57,D135,G55,G64 |
| *Cordyceps* | Cordycipitaceae | Hypocreales | Sordariomycetes | Ascomycota | KS23 |
| *Isaria* | Cordycipitaceae | Hypocreales | Sordariomycetes | Ascomycota | F61 |
| *Lecanicillium* | Cordycipitaceae | Hypocreales | Sordariomycetes | Ascomycota | F35,F84 |
| *Beauveria* | Cordycipitaceae | Hypocreales | Sordariomycetes | Ascomycota | E130,E37 |
| *Trichoderma*/*Hypocrea* | Hypocreaceae | Hypocreales | Sordariomycetes | Ascomycota | D142,E2,E15,G63,F43,JH12,JH27,JH45,LH12,Y2,DC23,DC13,HF23,KS70,LD19,QS7,QH12,QH36,QH46,QH59,QH8,QH83,QH9,QS16,RH1 |
| *Hirsutella*/*Ophiocordyceps* | Ophiocordycipitaceae | Hypocreales | Sordariomycetes | Ascomycota | G68 |
| *Stachybotrys* | Stachybotryaceae | Hypocreales | Sordariomycetes | Ascomycota | HF10 |
| *Myrothecium* | Incertae sedis | Hypocreales | Sordariomycetes | Ascomycota | L23,LD16 |
| *Sarocladium* | Incertae sedis | Hypocreales | Sordariomycetes | Ascomycota | HF2 |
| *Ilyonectria* | Incertae sedis | Hypocreales | Sordariomycetes | Ascomycota | D8,D139,F1,F38,X17,X39,X53,X85 |
| *Fimetariella* | Lasiosphaeriaceae | Sordariales | Sordariomycetes | Ascomycota | JH17 |
| *Podospora* | Lasiosphaeriaceae | Sordariales | Sordariomycetes | Ascomycota | BY37, KS74 |
| *Apodus* | Lasiosphaeriaceae | Sordariales | Sordariomycetes | Ascomycota | E77 |
| *Botryotrichum*/*Chaetomium* | Lasiosphaeriaceae | Sordariales | Sordariomycetes | Ascomycota | D16,D70,D76,D81,D98,E59,E62,E83,E84,E91,E97,G25,G35,F5,F17,F20,F33,JH5,JH20,QH51,QH98 |
| *Cladorrhinum*/*Cercophora* | Lasiosphaeriaceae | Sordariales | Sordariomycetes | Ascomycota | JH26,JH47 |
| *Schizothecium* | Lasiosphaeriaceae | Sordariales | Sordariomycetes | Ascomycota | KS50,RH18 |
| *Zopfiella* | Lasiosphaeriaceae | Sordariales | Sordariomycetes | Ascomycota | GZ31 |
| *Corynascella* | Chaetomiaceae | Sordariales | Sordariomycetes | Ascomycota | BY6 |
| *Chrysonilia*/*Neurospora* | Sordariaceae | Sordariales | Sordariomycetes | Ascomycota | JH7 |
| *Xylaria* | Xylariaceae | Xylariales | Sordariomycetes | Ascomycota | LH6,LD14,LD36 |
| *Geniculosporium*/*Nemania* | Xylariaceae | Xylariales | Sordariomycetes | Ascomycota | E46,LH3,LH10,CH11,QH58,DF54 |
| *Hypoxylon* | Xylariaceae | Xylariales | Sordariomycetes | Ascomycota | CH37 |
| *Nodulisporium*/*Daldinia* | Xylariaceae | Xylariales | Sordariomycetes | Ascomycota | HF11 |
| *Geniculisporium*/*Entoleuca* | Xylariaceae | Xylariales | Sordariomycetes | Ascomycota | LT51 |
| *Rosellinia* | Xylariaceae | Xylariales | Sordariomycetes | Ascomycota | DF13 |
| *Xylocoremium* | Xylariaceae | Xylariales | Sordariomycetes | Ascomycota | JH13 |
| *Annulohypoxylon* | Xylariaceae | Xylariales | Sordariomycetes | Ascomycota | DF43 |
| *Pestalotiopsis*/*Pestalosphaeria* | Amphisphaeriaceae | Xylariales | Sordariomycetes | Ascomycota | D121,LH17,ZG2,BY13,HF19,LD20,LD30,QS45,RH3 |
| *Truncatella*/*Broomella* | Amphisphaeriaceae | Xylariales | Sordariomycetes | Ascomycota | G46,GZ17,DC42 |
| *Libertella*/*Eutypella* | Diatrypaceae | Xylariales | Sordariomycetes | Ascomycota | E33,E35,E134,G23,YL7 |
| *Eutypella* | Diatrypaceae | Xylariales | Sordariomycetes | Ascomycota | LT15,LT36,QH27,QH40,QH41,QH92,DF18,DF14,DF62,DF50,DF48,DF46 |
| *Biscoqniauxia*/*Nodulisporium* | Graphostromataceae | Xylariales | Sordariomycetes | Ascomycota | E104,E105,HM10,JH2 |
| *Zygosporium* (*Geniculosporium*-like) | Zygosporiaceae | Xylariales | Sordariomycetes | Ascomycota | D101,D102,E101 |
| *Microdochium*/*Monographella* | Incertae sedis | Xylariales | Sordariomycetes | Ascomycota | E66,L2,X2,QS26,LT16,LT17,LT23,LT5,LT52 |
| *Pilidiella*/*Schizoparme* | Melanconidaceae | Diaporthales | Sordariomycetes | Ascomycota | D141 |
| *Phomopsis*/*Diaporthe* | Diaporthaceae | Diaporthales | Sordariomycetes | Ascomycota | D14,D28,D59,D125,D152,E132,E152,G77,F3,F4,F13,F16,F21,F22,F25,F50,F52,F54,F63,F71,F72,F77,F79,JH1,L26,L27,T10,T15,Y11,Z13,CH1,HF15,LD11,LD22,BY6,BY16,BY20,QH11,QH24,QH43,DF24 |
| *Pithoascus*/*Leptosphaerulina* | Microascaceae | Microascales | Sordariomycetes | Ascomycota | D147,D148,AH4,QS3,QS4 |
| *Sigmoidea*/*Corollospora* | Halosphaeriaceae | Microascales | Sordariomycetes | Ascomycota | D97 |
| *Phaeoseptoria*/*Phaeosphaeria* | Phaeochoraceae | Phyllachorales | Sordariomycetes | Ascomycota | DF11,KS62 |
| *Arthrinium* | Apiosporaceae | Incertae sedis | Sordariomycetes | Ascomycota | QS24,RH29,GZ81 |
| *Plectosporium*/*Plectosphaerella* | Plectosphaerellaceae | Incertae sedis | Sordariomycetes | Ascomycota | D5,D6,D13,D17,D26,D33,D36,D37,D38,D41,D43,D51,D52,D60,D66,D77,D90,D114,D120,D132,D144,D146,D150,E8,E9,E17,E21,E23,E24,E28,E31,E41,E45,E49,E50,E51,E54,E60,E69,E71,E72,E73,E78,E80,E82,E85,E88,E89,E94,E95,E100,E103,E108,E109,E113,E117,E123,E126,E129,E131,E133,E135,E136,E140,E142,E144,E146,G6,G40,G54,F2,F18,F29,F36,F37,F39,F45,F46,F47,F48,F65,F81,F85,F89,X6,X13,X44,HM9,JH8,JH21,JH23,JH31,JH48,L28,LH4,LH7,WB2,KS31,KS32,KS35,KS68,XG47,DF47,DF51,DF61,DF64,DF65,DF67,DF68,DF69,DF70 |
| *Colletotrichum*/*Glomerella* | Glomerellaceae | Glomerellales | Sordariomycetes | Ascomycota | D9,D12,D31,D34,D35,D108,D111,D151,D153,F6,F23,F26,F34,F56,F58,HM6,JH3,T1,T13,Y1,Y7,YL21,YL26,CH3,KS17,KS26,HF18,HF21,HF26,BY33,BY45,BY49,BY50,BY57,BY59,BY61,BY62,BY64,BY67,BY74,BY75,BY88,BY96,BY97,LT2,LT31,LT76,LT104,LT105,LT109,QH55,QH89 |
| *Nigrospora*/*Khuskia* | Incertae sedis | Incertae sedis | Sordariomycetes | Ascomycota | D84,D137,E4,E121,E125,X84,JH4,HX14,KS39,LD4,WX9,WX10,QH48,QH49 |
| *Phialemoniopsis* | Incertae sedis | Incertae sedis | Sordariomycetes | Ascomycota | DC25 |
| *Tricladium*/*Cudoniella* | Helotiaceae | Helotiales | Ascomycetes | Ascomycota | KS78 |
| *Varicosporium*/*Hymenoscyphus* | Helotiaceae | Helotiales | Ascomycetes | Ascomycota | X37,X75 |
| *Neofabraea* | Dermateaceae | Helotiales | Ascomycetes | Ascomycota | BY52 |
| *Leptodontidium* | Leptodontidiaceae | Helotiales | Ascomycetes | Ascomycota | BY43,DC35,LT80,LT88,LT89,LT90 |
| *Ciboria* | Sclerotiniaceae | Helotiales | Ascomycetes | Ascomycota | BY55 |
| *Botryosphaeria* | Botryosphaericeae | Pleosporales | Ascomycetes | Ascomycota | D4,G82,F19,F73 |
| *Sordaria* | Sordariaceae | Sphaeriales | Ascomycetes | Ascomycota | X21,X45 |
| *Phaeocytostroma* | Incertae sedis | Incertae sedis | Ascomycetes | Ascomycota | JH34 |
| *Periconia* | Incertae sedis | Incertae sedis | Ascomycetes | Ascomycota | E112,X9,X12,T9,WB4,BY12,GZ82,HX7,LT68,KS63,KS65,WX1,DF27 |
| *Talaromyces* | Aspergillaceae | Eurotiales | Eurotiomycetes | Ascomycota | Y9 |
| *Aspergillus*/Emericella,*Eurotium*,*Neosartorya* | Trichocomaceae | Eurotiales | Eurotiomycetes | Ascomycota | E22,E34,G28,G33,JH15,JH16,JH42,CH29,GZ5,GZ12,GZ15,GZ40,GZ42,GZ44,GZ46,GZ54,HF27,LD12,LT9,QH37,QH95,XG38,DF41 |
| *Merimbla* | Trichocomaceae | Eurotiales | Eurotiomycetes | Ascomycota | F80 |
| *Penicillium*/*Eupenicillium* | Trichocomaceae | Eurotiales | Eurotiomycetes | Ascomycota | E74,E122,F83,L17,BY14,DF61,GZ61,HX5,KS53,ZG5,QH85,QS41,XG88,DF20 |
| *Arthrobotrys*/*Orbilia* | Orbiliaceae | Helotiales | Leotiomycetes | Ascomycota | D27,X47,X130,LT70,QH47 |
| *Articulospora*/*Hymenoscyphus* | Helotiaceae | Helotiales | Leotiomycetes | Ascomycota | G67,X51,QS8,QS29,QS32 |
| *Botrytis*/*Botryotinia* | Sclerotiniaceae | Helotiales | Leotiomycetes | Ascomycota | GZ45,HX15,LT77 |
| *Geomyces*/*Pseudogymnoascus* | Myxotrichaceae | Helotiales | Leotiomycetes | Ascomycota | DC26,XG85 |
| *Pilidium*/*Discohainesia* | Chaetomellaceae | Helotiales | Leotiomycetes | Ascomycota | X11 |
| *Tetracladium* | Incertae sedis | Helotiales | Leotiomycetes | Ascomycota | RH28 |
| *Filosporella* | Incertae sedis | Helotiales | Leotiomycetes | Ascomycota | X87,X90,BY69,BY71,GZ72,GZ79,GZ90,QS5,QS14,QS17,QS30,XG20,LT24,LT92,LT94,DF16,DF36,DF38 |
| *Pleiochaeta* | Erysiphaceae | Erysiphales | Leotiomycetes | Ascomycota | DC39,DC52,XG50,XG67,XG71 |
| *Chromelosporium*(*Ostracoderma*) | Pezizaceae | Pezizales | Pezizomycetes | Ascomycota | E55,LH11,LH16 |
| *Peziza*(*Cyathus*) | Pezizaceae | Pezizales | Pezizomycetes | Ascomycota | L30 |
| *Pyronema* | Pyronemataceae | Pezizales | Pezizomycetes | Ascomycota | GZ65,RH4,LT113 |
| *Oedocephalum* | Cunninghamellaceae | Pezizales | Pezizomycetes | Ascomycota | G30 |
| *Meyerozyma* | Debaryomycetaceae | Saccharomycetales | Saccharomycetes | Ascomycota | DF39 |
| *Geotrichum*/*Dipodascus*,*Galactomyces* | Endomycetaceae | Saccharomycetales | Saccharomycetes | Ascomycota | YL1 |
| *Curvularia*/*Cochliobolus* | Pleosporaceae | Pleosporales | Euascomycetes | Ascomycota | D55,D75,G75 |
| *Acrostalagmus* | Hypocreaceae | Sphaeropsidales | FungiImperficti | Ascomycota | F86 |
| *Cytosporina*/*Eutypella* | Incertae sedis | Incertae sedis | Incertae sedis | Ascomycota | D15,E141,G31,JH6,L6,LH13,BY1,BY24,BY30,BY41,CH2,CH15,CH16,CH33,DF4,DF12,DC29,HF1,HF29,LD1,LD10,LD18,RH12,RH20,RH21,RH40,RH42 |
| *Scedosporium*/*Pseudallescheria* | Incertae sedis | Incertae sedis | Incertae sedis | Ascomycota | E64 |
| *Myrioconium*/*Sclerotinia* | Incertae sedis | Incertae sedis | Incertae sedis | Ascomycota | F42 |
| *Spegazzinia* | Incertae sedis | Incertae sedis | Incertae sedis | Ascomycota | HX8,HX9 |
| *Pseudeurotium* | Pseudeurotiaceae | Incertae sedis | Incertae sedis | Ascomycota | BY38,BY39,BY44,QS13,QS34,QS48,XG37 |
| *Heydenia* | Pyronemataceae | Pezizales | Pezizomycetes | Ascomycota | LD15,LD34,LD35 |
| *Dokmaia* (*Phaeoisaria*) | Incertae sedis | Incertae sedis | Incertae sedis | Ascomycota | L29 |
| *Virgariella* | Incertae sedis | Incertae sedis | Incertae sedis | Ascomycota | CH36,CH37,HX1,WX7 |
| *Irpex* | Meruliaceae | Polyporales | Agaricomycetes | Basidiomycota | D82,CH21,LT48 |
| *Phlebia* | Meruliaceae | Polyporales | Agaricomycetes | Basidiomycota | KS66 |
| *Perenniporia* | Polyporaceae | Polyporales | Agaricomycetes | Basidiomycota | HF8 |
| *Bjerkandera* | Polyporaceae | Polyporales | Agaricomycetes | Basidiomycota | D21,D23,X36,CH24,GZ53,LT102,DF17 |
| *Phaeophlebiopsis* | Polyporaceae | Polyporales | Agaricomycetes | Basidiomycota | QS20 |
| *Trametes* | Polyporaceae | Polyporales | Agaricomycetes | Basidiomycota | E63,BY4,BY21,LD33,DF9,HF16 |
| *Thermophymatospora*/*Ganoderma* | Polyporaceae | Polyporales | Agaricomycetes | Basidiomycota | E107 |
| *Phlebiopsis* | Phanerochaetaceae | Polyporales | Agaricomycetes | Basidiomycota | CH45 |
| *Porostereum* | Phanerochaetaceae | Polyporales | Agaricomycetes | Basidiomycota | QH44 |
| *Hormographiella*/*Coprinellus* | Psathyrellaceae | Agaricales | Agaricomycetes | Basidiomycota | D25,G4,X20,L22,CH20,CH35,KS73,LT84,QH31,QH93 |
| *Coprinopsis*(*Oudemansiella*) | Psathyrellaceae | Agaricales | Agaricomycetes | Basidiomycota | D72 |
| *Psathyrella* | Psathyrellaceae | Agaricales | Agaricomycetes | Basidiomycota | LH1,BY18,CH4,CH9,CH43,KS45,QH22,QH87,QS21,QS22,LD17 |
| *Schizophyllum* | Schizophyllaceae | Agaricales | Agaricomycetes | Basidiomycota | E53,G27,F78,L5,Y6,Y19 |
| *Clitopilus* | Entolomataceae | Agaricales | Agaricomycetes | Basidiomycota | HX17 |
| *Psilocybe* | Hymenogastraceae | Agaricales | Agaricomycetes | Basidiomycota | QH62 |
| *Hypholoma* | Strophariaceae | Agaricales | Agaricomycetes | Basidiomycota | DF37 |
| *Pholiota* | Strophariaceae | Agaricales | Agaricomycetes | Basidiomycota | DF44 |
| *Rhizoctonia*/*Thanatephorus* | Ceratobasidiaceae | Cantharellales | Agaricomycetes | Basidiomycota | LD27,XG65,QS27,DF29 |
| *Ceratobasidium* | Ceratobasidiaceae | Cantharellales | Agaricomycetes | Basidiomycota | XG81,XG82 |
| *Sistotrema* | Hydnaceae | Cantharellales | Agaricomycetes | Basidiomycota | QH56 |
| *Spiniger*/*Heterobasidion* | Bondarzewiaceae | Russulales | Agaricomycetes | Basidiomycota | QS12 |
| *Peniophora* | Peniophoraceae | Russulales | Agaricomycetes | Basidiomycota | E110,CH5,CH23,CH31,DC32,DC36,DC46,DC49,QS50,WX2,QH32 |
| *Echinodontium* | Echinodontiaceae | Russulales | Agaricomycetes | Basidiomycota | WX6 |
| *Fibularhizoctonia*/*Athelia* | Atheliaceae | Atheliales | Agaricomycetes | Basidiomycota | E30,E42,E116,L7,LT118,RH26 |
| *Hyphodontia* | Hymenochaetaceae | Hymenochaetales | Agaricomycetes | Basidiomycota | E6 |
| *Oliveorhiza*/*Oliveonia* | Oliveoniaceae | Auriculariales | Agaricomycetes | Basidiomycota | LH2 |
| *Necator*/*Phanerochaete* | Corticiaceae | Corticiales | Agaricomycetes | Basidiomycota | E7,E87,CH22,CH28,GZ20,QS11,QS23,LT101,DF26 |
| *Trichosporon*/*Trichosporonaceae* | Trichosporonaceae | Tremellales | Tremellomycetes | Basidiomycota | QH81,QH82 |
| *Dimorphospora*/*Hymenoscyphus* | Helotiaceae | Helotiales | FungiImperfecti | Basidiomycota | X46,DF30 |
| *Mortierella* | Mortierellaceae | Mortierellales | Mucoromycotina | Zygomycota | D117,T12,Z7,ZG4,YL11,GZ7,GZ27,LT4,LT54,LT55 |
| *Rhizopus* | Mucoraceae | Mucorales | Zygomycetes | Zygomycota | E98,LT34,LT42 |

**TABLE S4.** Isolation frequency, the number of genera and diversity of endophytic fungi calculated from thirty aquatic plants collected from Southwest China.

| **Host Plants** | **Root Isolation Frequency** | **Stem Isolation Frequency** | **Leaf Isolation Frequency** | **Total Isolation Frequency** | **No. of Genera** | **H′** | **Evenness** |
| --- | --- | --- | --- | --- | --- | --- | --- |
| *A. philoxeroides* | 20.7%  (6/29) | 15.6%  (14/90) | 22.7%  (17/75) | 19.1%  (37/194) | 11 | 3.11 | 0.89 |
| *B. bungei* | 8.7%  (52/596) | 10.1%  (56/552) | 6.5%  (47/718) | 8.3%  (155/1866) | 29 | 3.78 | 0.79 |
| *C. demersum* | N/A | 14.4%  (12/85) | 5.8%  (15/257) | 7.9%  (27/342) | 20 | 4.25 | 0.98 |
| *Eg. densa* | N/A | 10%  (1/10) | 10%  (1/10) | 10.0%  (2/20) | 1 | —— | —— |
| *Ei. crassipes* | 13.4%  (15/112) | 8.0%  (14/176) | 11.5%  (21/182) | 17.7%  (56/317) | 6 | 2.23 | 0.86 |
| *E. canadensis* | 7.4%  (5/68) | 2%  (7/135) | 9.5%  (20/210) | 7.7%  (32/413) | 9 | 2.95 | 0.93 |
| *Hi. vulgaris* | 9.3%  (85/917) | 12.7%  (127/1001) | 18.1%  (168/926) | 13.2%  (380/2874) | 48 | 4.20 | 0.76 |
| *Hy. verticillata* | 4.2%  (2/48) | 10.7%  (12/112) | 8.5%  (28/329) | 10.3%  (42/409) | 13 | 3.22 | 0.87 |
| *H. dubia* | 18.4%  (7/38) | 18.6%  (8/43) | 24%  (12/50) | 22.3%  (27/121) | 13 | 3.7 | 1 |
| *I. sinensis*1 | 13.7%  (10/73) | 6.1%  (2/33) | 22.6%  (14/62) | 15.5%  (26/168) | 6 | 2.20 | 0.95 |
| *I. sinensis*2 | 16.7%  (8/48) | N/A | 19.4%  (12/62) | 18.2%  (20/110) | 10 | 3.28 | 0.99 |
| *Me. sativa* | N/A | N/A | 9.4%  (3/32) | 9.4%  (3/32) | 2 | 0.92 | 0.92 |
| *M. aquaticum* | 7.1%  (1/14) | 5.9%  (1/17) | 23.1%  (3/13) | 11.4%  (5/44) | 3 | 1.5 | 0.95 |
| *M. sibiricum* | 27.3%  (18/66) | 6.1%  (10/163) | 11.5%  (10/87) | 12.0%  (38/316) | 16 | 3.76 | 0.94 |
| *M. spicatum* | 13.1%  (71/541) | 9.8%  (126/1288) | 11.3%  (129/1145) | 11.0%  (326/2974) | 75 | 5.40 | 0.87 |
| *N. peltatum* | 2.2%  (1/46) | 5.9%  (1/17) | 5.8%  (3/52) | 5.2%  (6/115) | 4 | 2 | 1 |
| *O. acuminata* | N/A | N/A | 16.7%  (5/30) | 14.0%  (7/50) | 6 | 2.59 | 1 |
| *Pi. stratiotes* | 7.5%  (3/40) | 12.6%  (24/190) | 3.9%  (5/128) | 11.3%  (32/282) | 5 | 2.32 | 1 |
| *Po. amphibium* | N/A | 17.4%  (4/23) | 50%  (4/8) | 25.8%  (8/31) | 3 | 1.25 | 0.79 |
| *P. acutifolius* | N/A | 9.1%  (4/44) | 20%  (5/25) | 13.0%  (9/69) | 4 | 1.92 | 0.96 |
| *P. distinctus* | 12.6%  (51/404) | 15.0%  (69/459) | 5.4%  (13/241) | 16.7%  (186/1111) | 21 | 4.02 | 0.93 |
| *P. intortifolius* | 27.3%  (3/11) | 10.6%  (5/47) | 14.3%  (4/28) | 14.0%  (12/86) | 22 | 1.67 | 0.72 |
| *P. lucens* | N/A | 13.0%  (6/46) | 11.9%  (7/59) | 11.0%  (13/118) | 5 | 2 | 0.86 |
| *P. oxyphyllus* | 7.7%  (7/91) | 6.2%  (11/177) | 12.3%  (13/106) | 8.3%  (31/374) | 4 | 1 | 1 |
| *P. pectinatus* | 8.7%  (25/288) | 8.1%  (35/432) | 7.9%  (31/394) | 8.2%  (91/1114) | 29 | 4.32 | 0.90 |
| *P. perfoliatus* | 5.5%  (4/70) | 6.7%  (20/297) | 8.6%  (22/257) | 7.4%  (46/624) | 25 | 4.51 | 0.96 |
| *P. wrightii* | 16.0%  (7/44) | 11.1%  (26/235) | 9.8%  (27/276) | 10.4%  (60/575) | 28 | 4.70 | 0.99 |
| *S. trifolia* | 7.1%  (1/14) | N/A | 42.9%  (6/14) | 25.0%  (7/28) | 4 | 2 | 1 |
| *T. bispinosa* | 13.7%  (7/51) | 8.0%  (14/176) | 11.5%  (21/182) | 10.3%  (42/409) | 4 | 1.92 | 0.96 |
| *T. natans* | 66.7%  (4/6) | 50%  (4/8) | 66.7%  (4/6) | 60.0%  (12/20) | 8 | 2.95 | 0.98 |
| *V. natans* | 13.0%  (9/69) | 6.7%  (1/15) | 10.8%  (9/83) | 11.4%  (19/167) | 8 | 2.56 | 0.85 |
| All plants | 10.7%  (387/3604) | 10.5%  (607/5769) | 11.5%  (662/5762) | 11%  (1697/15373) | 158 | 5.23 | 0.72 |

‘N/A’ indicates that isolate was not available from the tissue segments.

When the number of plant tissue segments analyzed was over 100, the isolation frequency and diversity index among samples were more reliable.

**TABLE S5.** Isolation frequency, the number of genera and diversity of endophytic fungi calculated from different sites in Southwest China.

| **Sampling sites** | **Root Isolation Frequency** | **Stem Isolation Frequency** | **Leaf Isolation Frequency** | **Total Isolation Frequency** | **No. of Genera** | **H′** | **Evenness** |
| --- | --- | --- | --- | --- | --- | --- | --- |
| Dianchi | 14.00%  (46/328) | 17.3%  (62/359) | 7.90%  (20/252) | 13.60%  (128/939) | 42 | 4.43 | 0.83 |
| Erhai | 13.60%  (22/162) | 16.10%  (57/353) | 15.10%  (64/425) | 15.20%  (143/940) | 37 | 3.95 | 0.76 |
| Fuxianhu | 17.10%  (14/82) | 18.60%  (33/177) | 21.50%  (40/186) | 19.60%  (87/445) | 25 | 3.86 | 0.83 |
| Jianhu | 8.90%  (7/79) | 8.40%  (18/214) | 10.50%  (25/239) | 9.40%  (50/532) | 20 | 4.05 | 0.94 |
| Lashihai | N/A | 15.70%  (14/89) | 17.80%  (19/107) | 15.30%  (33/216) | 19 | 4.14 | 0.97 |
| Wenbihai | N/A | N/A | 11.80%  (4/34) | 11.80%  (4/34) | 2 | 1.00 | 1.00 |
| Heilongtan | N/A | 11.80%  (9/76) | 9%  (9/100) | 10.20%  (18/176) | 12 | 3.51 | 0.98 |
| Huamajie | 18.20%  (2/11) | 7.00%  (4/57) | 11.90%  (8/67) | 10.40%  (14/135) | 8 | 2.99 | 0.99 |
| Yangzonghai | 10.70%  (3/28) | 11.40%  (4/35) | 13.00%  (6/46) | 11.90%  (13/109) | 9 | 3.06 | 0.96 |
| Tengchong | 17.90%  (5/28) | 11.10%  (2/18) | 8.16%  (4/49) | 11.60%  (11/95) | 6 | 2.5 | 0.97 |
| Beimiao | N/A | 15.40%  (2/13) | 8%  (2/25) | 10.50%  (4/38) | —— | —— | —— |
| Banqiao | 7.70%  (1/13) | N/A | N/A | 7.70%  (1/13) | —— | —— | —— |
| Xianggelila | 16.70%  (83/500) | 17.50%  (85/486) | 11.20%  (44/393) | 15.40%  (212/1379) | 27 | 4.28 | 0.90 |
| Yila | 7.20%  14/194 | 7.30%  (9/124) | 8.20%  (6/73) | 7.40%  (29/391) | 8 | 2.59 | 0.86 |
| Gongkahu | 9.50%  (12/126) | 12.20%  (35/288) | 15.50%  (37/238) | 12.90%  (84/652) | 23 | 3.45 | 0.76 |
| Xiajisha | 13.70%  (10/73) | 6.10%  (2/33) | 22.60%  (14/62) | 15.50%  (26/168) | 6 | 2.16 | 0.84 |
| Chaohai | 7.70%  (7/91) | 8.40%  (25/296) | 12.30%  (19/154) | 9.40%  (51/541) | 18 | 3.98 | 0.96 |
| Huaxi | 21.40%  (3/14) | 4.10%  (11/179) | 4.40%  (17/197) | 5.40%  (11/390) | 13 | 3.66 | 0.99 |
| Hongfenghu | 15%  (3/20) | 12%  (6/50) | 18.70%  (20/107) | 16.40%  (29/177) | 12 | 3.46 | 0.96 |
| Ahahu | N/A | 7.70%  (2/26) | 4.10%  (2/49) | 5.30%  (4/75) | 1 | —— | —— |
| Qionghai | 10.10%  (22/217) | 6.90%  (23/335) | 10.60%  (49/464) | 9.30%  (94/1016) | 25 | 4.24 | 0.91 |
| Luding | 13.70%  (7/51) | 6.90%  (11/160) | 11.40%  (19/167) | 9.80%  (37/378) | 16 | 3.86 | 0.97 |
| Ruogaier | 4.20%  (9/215) | 8.20%  (21/255) | 8.40%  (19/227) | 7.00%  (49/697) | 14 | 3.52 | 0.92 |
| Litang | 12.70%  (62/512) | 10.30%  (60/582) | 11.70%  (64/549) | 11.30%  (186/1643) | 25 | 3.29 | 0.71 |
| Qisehai | 14.50%  (9/62) | 8.80%  (17/192) | 3.00%  (15/165) | 9.80%  (41/419) | 17 | 3.91 | 0.96 |
| Wuxuhai | 3.40%  (2/59) | 2.70%  (6/224) | 1.80%  (3/168) | 2.40%  (11/451) | 6 | 2.50 | 0.97 |
| Daofu | 11.0%  (19/172) | 8.5%  (29/343) | 6.1%  (22/363) | 8.0%  (80/878) | 29 | 4.40 | 0.91 |
| Kashahu | 8.60%  (25/294) | 10.70%  (28/262) | 11.70%  (25/214) | 10.10%  (78/770) | 22 | 3.68 | 0.83 |
| Daocheng | N/A | 13.10%  (23/175) | N/A | 13.10%  (23/175) | 12 | 2.73 | 0.76 |
| Baiyu | 9.00%  (25/277) | 10.80%  (37/344) | 13.60%  (44/324) | 11.20%  (106/945) | 21 | 3.63 | 0.83 |
| Ganzi | 10.60%  (32/301) | 4.10%  (15/363) | 9.20%  (43/466) | 8.00%  (90/1130) | 20 | 3.81 | 0.88 |

‘N/A’ indicates no data or no results.

When the number of plant tissue segments analyzed was over 100, the isolation frequency and diversity index among samples were more reliable.
